# Trapping an octahedral Ag_6_ kernel in a seven-fold symmetric Ag_56_ nanowheel

**DOI:** 10.1038/s41467-018-04499-9

**Published:** 2018-05-29

**Authors:** Zhi Wang, Hai-Feng Su, Mohamedally Kurmoo, Chen-Ho Tung, Di Sun, Lan-Sun Zheng

**Affiliations:** 1 Key Laboratory of the Colloid and Interface Chemistry, Ministry of Education, and School of Chemistry and Chemical Engineering, Shandong University, 250100 Jinan, China; 2 State Key Laboratory for Physical Chemistry of Solid Surfaces and Department of Chemistry, College of Chemistry and Chemical Engineering, Xiamen University, 361005 Xiamen, China; 3 Institut de Chimie de Strasbourg, Université de Strasbourg, CNRS-UMR 7177, 4 Rue Blaise Pascal, 67008 Strasbourg Cedex, France

## Abstract

High-nuclearity silver clusters are appealing synthetic targets for their remarkable structures, but most are isolated serendipitously. We report here six giant silver-thiolate clusters mediated by solvents, which not only dictate the formation of an octahedral Ag_6_^4+^ kernel, but also influence the in situ-generated Mo-based anion templates. The typical sevenfold symmetric silver nanowheels show a hierarchical cluster-in-cluster structure that comprises an outermost Ag_56_ shell and an inner Ag_6_^4+^ kernel in the centre with seven MoO_4_^2−^ anion templates around it. Electrospray ionization mass spectrometry analyses reveal the underlying rule for the formation of such unique silver nanowheels. This work establishes a solvent–intervention approach to construct high-nuclearity silver clusters in which both the formation of octahedral Ag_6_^4+^ kernel and in situ generation of various Mo-based anion templates can be simultaneously controlled.

## Abstract (web summary)

High-nuclearity silver clusters are appealing synthetic targets for their remarkable structures, but most are isolated serendipitously. Here, the authors describe the rational use of solvents to form cluster-in-cluster silver nanowheels, which comprise an octahedral Ag_6_^4+^ core surrounded by a Ag_56_ cage of unusual seven-fold symmetry.

## Introduction

High-nuclearity clusters of silver ions continue to fascinate chemists because of their intriguing geometrical characteristics such as high symmetry, large dimension and architectural beauty as well as some promising applications^1–3^. Recent advances in the silver cluster field clearly show the subdivisions: type-I, the silver atoms exclusively aggregate to the cluster core and show an average oxidation state between 0 and 1^4–9^; type-II, the formation of metal core is not exclusively from the aggregation of monovalent silver but with the participation of small anions or/and organic ligands^10–16^. The representatives of type-I and -II clusters are [Ag_44_(*p*-MBA)_30_]^4^^−^^17^ and [Ag_490_S_188_(S*t*C_5_H_11_)_114_]^18^, respectively. These two kinds of silver clusters may be seen as molecular embryos of the bulk phases of silver metal and binary silver sulfides, respectively, and thus emerged as a hot frontier in chemistry and nanoscience research.

We have been working on the development of new strategies to access silver clusters with some level of control on the metal nuclearity and geometry for a given ligand type^19–24^. However, we must admit that almost all silver clusters were originally isolated serendipitously and the cases of deliberate modulation of nuclearity and geometry are very few, with most based on an anion template method. Among diverse anions, polyoxometalates (POMs) as a family of inorganic cluster have very rich structures and interesting properties^25–31^. Recent work showed the encapsulation of POMs into silver-thiolate/alkynyl clusters could effectively direct the nuclearity and geometry of the final products depending on the size and shape of the POMs ^ 32–38^. Moreover, POMs usually isomerize or transform to other forms different from their original compositions and structures when enwrapped into the silver clusters^39^, which should be controlled by the pH of the assembly environment and influenced by polarity of solvents^40^. On the other hand, solvents such as DMF (*N*,*N*-Dimethylformamide) contain aldehyde group that not only could endow electrons from O atom to form coordination bond with metal centre, but also has the ability to reduce Ag(I) to Ag(0)^41^, which favours the formation of type-I silver clusters.

With these points in mind, herein, we show that it is quite effective to follow the solvent–intervention strategy to combine two types of silver clusters into one compound. They are sevenfold symmetry cluster-in-cluster silver nanowheels with an octahedral Ag_6_^4+^ kernel trapped in the centre. The family of **SD/Ag7**–**SD/Ag10** (**SD** = **SunDi**) is the highest-nuclearity silver clusters possessing wheel-like topology and trapping the maximum MoO_4_^2−^ ions as template in one wheel. To justify the unique solvent effect in this assembly system, another two high-nuclearity silver-thiolate clusters (**SD/Ag11** and **SD/Ag12**) are isolated with the Ag_6_^4+^ kernel template replaced by two novel POMs in the absence of DMF. This work pioneers a solvent–intervention approach to construct high-nuclearity silver nanowheels incorporating both an octahedral Ag_6_^4+^ kernel and a sevenfold symmetric silver shell.

## Results

### Structures of SD/Ag7–SD/Ag10

**Fig. 1:**
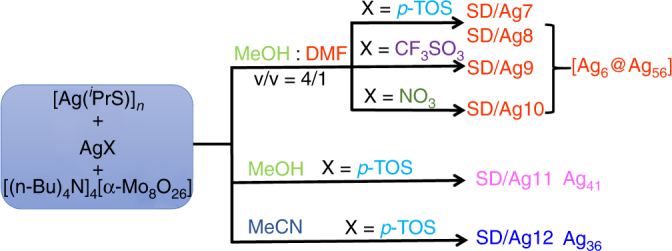
Schematic representation of the synthesis of clusters **SD/Ag7**-**SD/Ag/12**. These clusters are formed by a solvent-controlled method

Although *p*-TOS^−^, CF_3_SO_3_^−^ and NO_3_^−^ were employed as anions in the construction of **SD/Ag7–SD/Ag10** from CH_3_OH/DMF (v:v = 4:1) as a binary solvent (Fig. 1), single-crystal X-ray diffraction (SCXRD) analyses showed that their sevenfold symmetric wheel-like topology was conserved, suggesting these anions have little effects on the cluster skeletons. Due to different molecules packing in the lattice (Supplementary Figure 1), they crystallize in different space groups, monoclinic *P*2_1_/*c*, monoclinic *C*2/*c*, tetragonal *P*4_2_/*ncm* and monoclinic *I*2/*m* space groups, respectively. Their formulae were determined as [Ag_6_@(MoO_4_)_7_@Ag_56_(MoO_4_)_2_(^*i*^PrS)_28_(*p*-TOS)_14_(DMF)_4_] (**SD/Ag7**), [Ag_6_@(MoO_4_)_7_@Ag_56_(MoO_4_)_2_(^*i*^PrS)_28_(*p*-TOS)_14_·3DMF] (**SD/Ag8**), [Ag_6_@(MoO_4_)_7_@Ag_56_(MoO_4_)_2_(^*i*^PrS)_28_(CF_3_SO_3_)_14_(DMF)_4_] (**SD/Ag9**), [Ag_6_@(MoO_4_)_7_@Ag_56_(MoO_4_)_2_(^*i*^PrS)_28_(NO_3_)_14_] (**SD/Ag10**). Because of the striking similarities of the molecules in **SD/Ag7–SD/Ag10**, only that of **SD/Ag7** is described in detail here. Selected details of the data collection and structure refinements are listed in Supplementary Table 1.

**Fig. 2:**
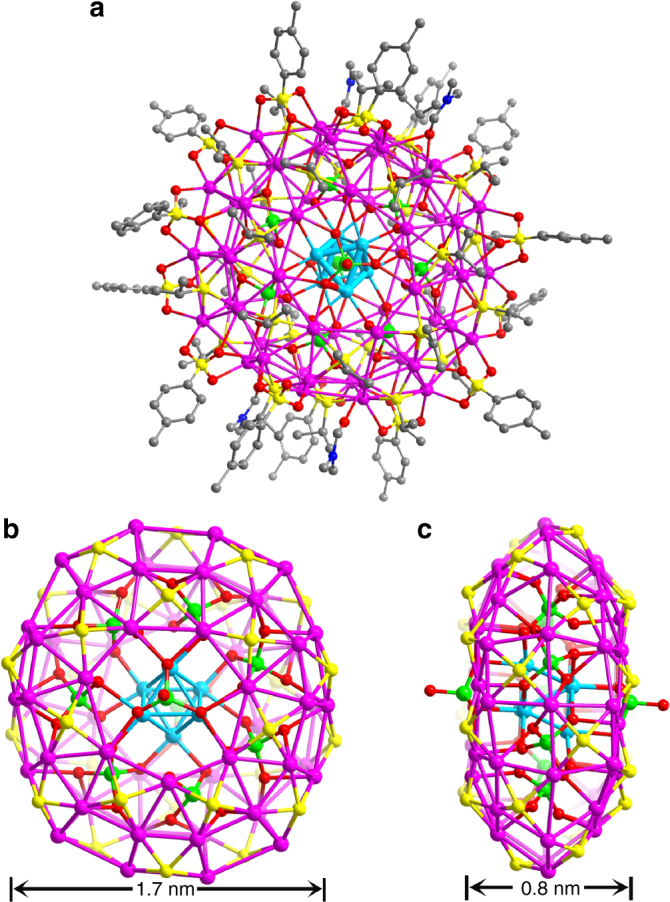
Single-crystal structure of **SD/Ag7**. **a** The total structure of **SD/Ag7** showed in ball-and-stick model. Ball-and-stick model of **SD/Ag7** showing the front (**b**) and side (**c**) views with all C atoms omitted for clarity. (Colour legend: purple, Ag on the shell; cyan, Ag in the kernel; yellow, S; red, O; green, Mo)

The centrosymmetric silver wheel of **SD/Ag7**, as shown in Fig. 2a, like a motorcycle tyre, comprises of an Ag_56_ shell and an octahedral Ag_6_^4+^ kernel in the centre with up to seven MoO_4_^2−^ as anion templates around it. Two additional MoO_4_^2−^ anions serve as hubcaps of the wheel. This wheel has a large dimension even without considering the organic ligands, measuring ~1.7 nm across its circular face (Fig. 2b) and 0.8 nm across the rectangular rim (Fig. 2c). It resembles the Preyssler-type polyoxometalate. In an asymmetric unit, a half of a wheel was identified and other half could be generated by inversion symmetry. The Ag_56_ shell is covered by 28 ^*i*^PrS^−^ and 14 *p*-TOS^−^ ligands and consolidated by abundant Ag⋯Ag interactions (2.926–3.367 Å), forming 14 silver trigons and 42 silver tetragons fused together in an edge-sharing manner. In detail, on the circular face of the wheel, the silver trigons are isolated by silver tetragons and share their edges; whereas all silver tetragons are fused together to form the rectangular rim of the wheel (Supplementary Figure 2). All ligands exclusively capped on the tetragonal faces, where µ_4_^*i*^PrS^−^, µ_4_−η^2^:η^1^:η^1^ *p*-TOS^−^ and µ_3_−η^1^:η^1^:η^1^ *p*-TOS^−^ were observed with the Ag–S and Ag–O distances ranging 2.374–2.552 and 2.400–2.770 Å, respectively.

**Fig. 3:**
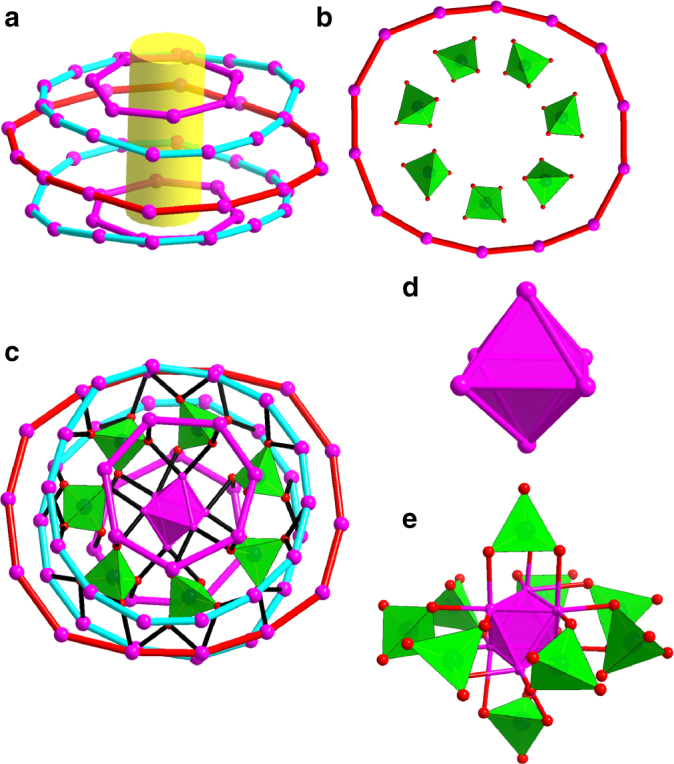
Disassembled skeletal structure of **SD/Ag7**. **a** Five concentric metallocycles: two Ag_7_ (purple); two small Ag_14_ (cyan) and one large Ag_14_ (red). **b** Seven MoO_4_^2−^ anions arranged along the equatorial Ag_14_ ring. **c** The bonding of MoO_4_^2−^ with Ag_14_ rings and inner Ag_6_ kernel with Ag–O bonding highlighted by black. **d** The polyhedral mode showing octahedral Ag_6_ kernel. **e** The Ag_6_ kernel protected by nine MoO_4_^2−^ anions with seven in the equator and two at the poles. (Colour legend: purple, Ag; red, O; green, Mo)

Alternatively, we can also imagine this wheel as five concentric metallocycles of different diameters linked by µ_4_^*i*^PrS^−^ ligands. The five metallocycles, nearly perpendicular to the pseudo sevenfold axis, contain one large Ag_14_ ring at the equator (*ϕ* = 17.14 Å; *d*_Ag__⋯__Ag_ = 3.554–4.032 Å) with two Ag_7_ rings (*ϕ* = 7.68 Å; *d*_Ag__⋯__Ag_ = 3.366–3.797 Å) and two small Ag_14_ rings (*ϕ* = 13.66 Å; *d*_Ag__⋯__Ag_ = 2.926–3.331 Å) lying above and below this ring (Fig. 3a). Two Ag_7_ rings are not face-to-face stacking but rotated by an angle of 27.6° with respect to each other, whereas two small Ag_14_ rings are almost face-to-face stacking (Supplementary Figure 3). Interestingly, we found that up to seven MoO_4_^2−^ anions were arranged along the equatorial Ag_14_ ring inside (Fig. 3b). All seven MoO_4_^2−^ ions adopt the same μ_8_−η^2^:η^2^:η^2^:η^2^ bonding fashion to all Ag_14_ rings as well as the inner octahedral Ag_6_^4+^ kernel with an average Ag–O distance of 2.444 Å (Fig. 3c). This is the maximum number of MoO_4_^2−^ ions as template trapped in one cluster.  The previous record was a 55-nuclearity silver-alkynyl cluster with six MoO_4_^2−^ ions inside^42^. Not only the number of Ag atoms in each ring, but also the number of MoO_4_^2−^ ions are 7 or multiples of 7, which ultimately leads to, albeit not rigorous, a sevenfold symmetry of the overall wheel. The overall geometry of such cluster shows typical anisotropy with rare high-order odd symmetry which should be dictated by initially prearranged seven MoO_4_^2−^ anions around Ag_6_^4+^ kernel, as justified by a series of [Ag_6_@(MoO_4_)_7_@Ag_*n*_] (*n* = 28–32) intermediates observed in the electrospray ionization mass spectrometry (ESI-MS) of an early-stage reaction mixture during the synthesis of **SD/Ag7** (Supplementary Figure 4 and Supplementary Table 2). Generally, the orders of symmetry with a prime number equal to or higher than 5 are disfavoured^43^, thus artificial molecular clusters with five- or sevenfold symmetry remain a rarity^44–46^, although such symmetries usually exist in biomacromolecules such as HslV (heat shock locus V) and chaperone GroEL^47, 48^. The sevenfold symmetric characteristic is the first observed in silver clusters as exemplified by **SD/Ag7**–**SD/Ag10**.

Another structural feature is a centremost octahedral Ag_6_^4+^ kernel (Ag1, Ag2, Ag3 and their symmetry equivalents) which is formed purely by Ag⋯Ag interactions ranging from 2.659 to 2.852 Å (Fig. 3d). These short contacts between silver atoms are even shorter than those in bulk Ag metal (2.886 Å), indicating the significant argentophilicity^49–51^. The Ag_6_ kernel is stabilized by a total of nine MoO_4_^2−^ anions through the Ag–O bonding (Fig. 3e) at the exposed [111] facets or edges of octahedron. This Ag_6_^4+^ kernel can be deemed as the smallest single-crystal octahedral silver nanocrystal^52^ cut from the face-centred cubic (*fcc*) lattice of bulk silver but with a slight shrinkage due to the addition of two more electrons to the bonding orbitals^53^. Although several inorganic compounds, such as Ag_6_Ge_10_P_12_^54^, Ag_6_O_2_^55^, Ag_5_SiO_4_^56^ and Ag_5_GeO_4_^57^, have been identified to contain such subvalent cluster unit, it is still rarely found in ligand-capped silver clusters^58, 59^. The formation of such partially reduced species should be caused by the reductive ability of DMF, which is widely used in the controlled synthesis of multiple-twin decahedral and icosahedral silver nanocrystals with special favourable [111] faces by reducing Ag^+^ to Ag^0^^41^. During this assembly process, DMF was partially oxidized to Me_2_NCOOH, which was unambiguously verified by the ^13^C NMR (nuclear magnetic resonance) of HCl-digested reaction mother solution (Supplementary Figure 5). Based on above analyses and discussions, we found the reducing ability of DMF dictates the formation of the innermost Ag_6_^4+^ kernel, which then attracts seven MoO_4_^2−^ anions around it, thus forming the final sevenfold symmetric silver nanowheel. All these results clearly demonstrate that the reducibility of DMF is the key to such unique silver nanowheel.

Molecular wheels caught chemists’ eyes as far back as 20-year ago since the giant POM wheels (Mo_154_, Mo_176_ and even larger Mo_248_) were first characterized by Müller using X-ray diffraction^60^. Following their work, other giant wheel-like clusters, such as Mn_84_^61^, Ni_24_^62^ and Pd_84_^63^, were also successfully synthesized, suggesting such clusters were possible beyond Mo-based POMs. Among them, Mo_154_ and Pd_84_ are two limited sevenfold symmetric wheels. The wheel-like topology in silver clusters has not been reported up to now.

A careful examination of the packing of the wheels in the crystal lattice of **SD/Ag7** reveals that there are intermolecular C–H⋯O hydrogen bonding and van der Waals force between neighbours; the nearest Ag⋯Ag distance between neighbouring units is ~8.3 Å, with nearest neighbours oriented parallel to one another, that is, packing face to face (Supplementary Figure 1). However, the wheels packing showed an inclined rim-to-face orientation for **SD/Ag8** and **SD/Ag9**, and a displaced face-to-face orientation for **SD/Ag10**.

### Structures of SD/Ag11 and SD/Ag12

The comparative experiment to justify the reduction-induced formation of subvalent Ag_6_^4+^ kernel has resulted in the isolation of another two huge silver clusters **SD/Ag11** and **SD/Ag12** in the absence of DMF ([Mo_7_O_24_@Ag_41_(^*i*^PrS)_19_(*p*-TOS)_16_(CH_3_OH)_4_·4CH_3_OH] (**SD/Ag11**) and (*n*-Bu_4_N)_1.5_[Mo_5_O_18_@Ag_36_(^*i*^PrS)_18_(*p*-TOS)_13.5_(CH_3_CN)·1.5CH_3_CN] (**SD/Ag12**)). In their centres, two novel POMs anions are trapped instead of the Ag_6_^4+^ kernel, suggesting the decisive role of DMF on the formation of subvalent Ag_6_^4+^ kernel.

**Fig. 4:**
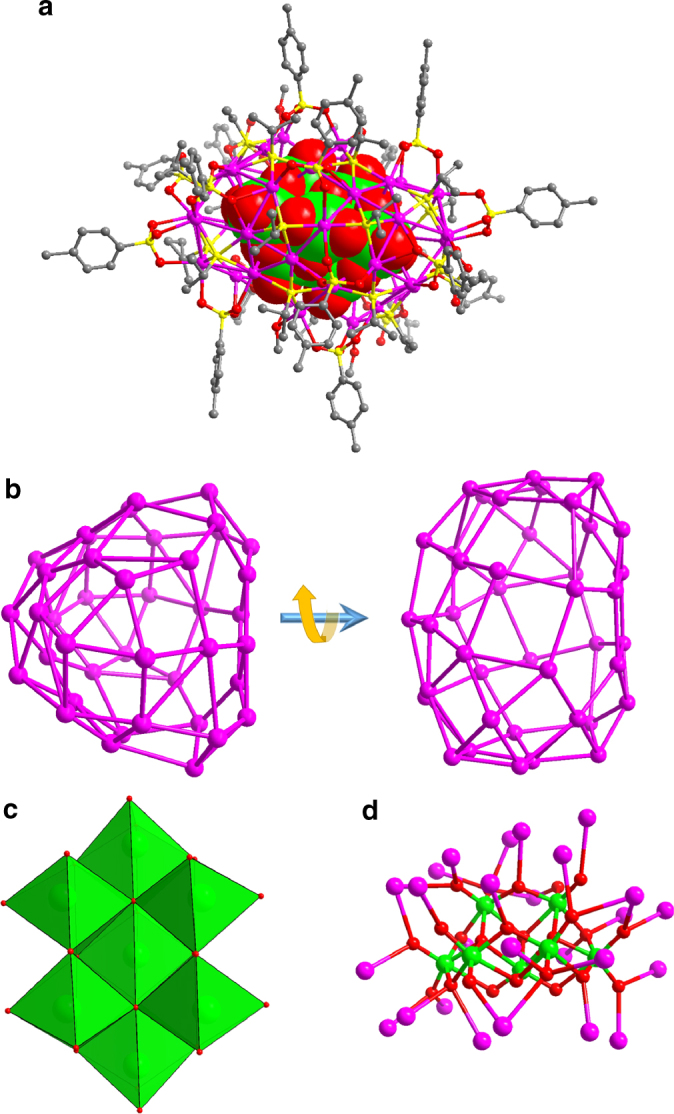
Single-crystal structure of **SD/Ag11**. **a** The total structure of **SD/Ag11** with Mo_7_O_24_^6−^ shown in space filling mode. **b** The Ag_41_ skeleton viewed along different directions. **c** Polyhedral representation of Mo_7_O_24_^6−^. **d** The bonding between Mo_7_O_24_^6−^ and Ag atoms at the shell. (Colour legend: purple, Ag; yellow, S; red, O; green, Mo)

When the solvent was changed to methanol, **SD/Ag11** crystallized in the orthorhombic space group *Pccn* and was isolated as a 41-nuclearity ellipsoidal silver shell loaded with a *C*_2v_-Mo_7_O_24_^6−^ in the centre as template (Fig. 4a). There are 19 μ_4_^*i*^PrS^−^, 16 *p*-TOS^−^ (2 × μ_4_−η^2^:η^1^:η^1^, 6 × μ_3_−η^1^:η^1^:η^1^, 6 × μ_2_−η^1^:η^1^ and 2 × μ_4_−η^2^:η^2^), four CH_3_OH (2 × μ_2_ and 2 × μ_1_) molecules coordinated on this irregular Ag_41_ shell with the Ag–S and Ag–O distances of 2.388–2.532 and 2.338–2.592 Å. The abundant Ag⋯Ag interactions (2.886–3.374 Å) finally reinforce the overall shell (Fig. 4b). As shown in Fig. 4c, an Mo_7_O_24_^6−^ ion was clearly resolved and the bond valence sum (BVS) calculations^64^ indicate that all Mo atoms have an oxidation state of +6 (Mo1: 5.811; Mo2: 5.716; Mo3: 6.002 and Mo4: 6.030). All Mo atoms are coordinated to six O atoms, forming seven MoO_6_ octahedra, which are fused to Mo_7_O_24_^6−^ by exclusively edge-sharing fashion. The Mo–O bond lengths vary from 1.711 to 2.511 Å. The 41 silver atoms arranged around the surface of Mo_7_O_24_^6−^ forming the core–shell structure. Among 41 silver atoms, 26 are coordinated to both terminal and bridge O atoms (Fig. 4d) with Ag–O distances in the range of 2.378–2.783 Å. The Mo_7_O_24_^6–^ should be in situ generated from α-Mo_8_O_26_^4−^.

**Fig. 5:**
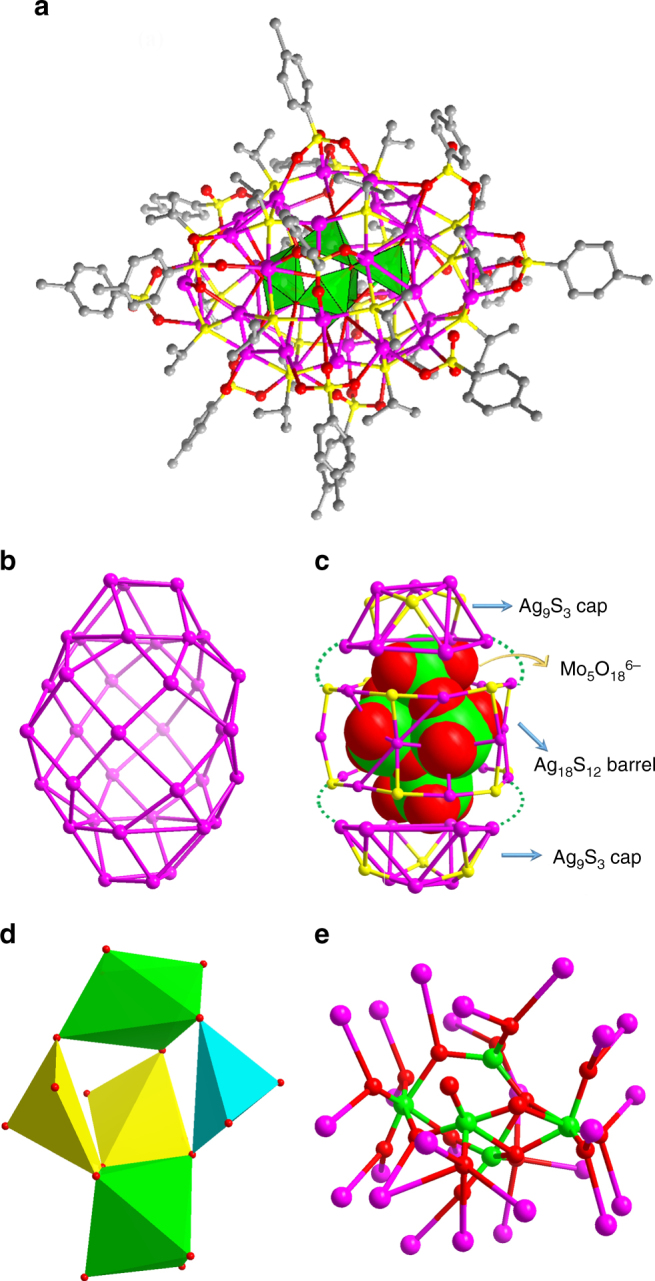
Single-crystal structure of **SD/Ag12**. **a** The total structure of **SD/Ag12** with Mo_5_O_18_^6−^ shown in polyhedral mode. **b** The Ag_36_ skeleton comprised of Ag_3_ trigons and Ag_4_ tetragons. **c** Schematic showing one Mo_5_O_18_^6−^ enwrapped by Ag_18_ barrel, then closed by two half-cuboctahedral Ag_9_S_3_ caps. **d** Polyhedral representation of Mo_5_O_18_^6−^ with two octahedral MoO_6_, two square-pyramidal MoO_5_ and one tetrahedral MoO_4_ units shown in green, yellow and cyan, respectively. **e** The bonding between Mo_5_O_18_^6−^ and Ag atoms at the shell. (Colour legend: purple, Ag; yellow, S; red, O; green, Mo)

When using CH_3_CN as solvent, we isolated **SD/Ag12** as a 36-nuclearity silver cluster with an Mo_5_O_18_^6−^ as core as revealed by SCXRD structural analysis (Fig. 5a). This anionic Ag_36_ cluster can be shaped as a rugby-like skeleton (Fig. 5b) built from an equatorial Ag_18_S_12_ barrel adding two half-cuboctahedral Ag_9_S_3_ caps above and below, and they are joined together by argentophilic interactions and through Ag–S bonds (Fig. 5c). The Ag_36_ shell is peripherally bridged by 18 μ_4_^*i*^PrS^−^, 13.5 *p*-TOS^−^ (9 × μ_3_−η^1^:η^1^:η^1^, 1 × μ_3_−η^2^:η^1^ and 3.5 × μ_2_−η^1^:η^1^) and one CH_3_CN molecule. The Ag–S and Ag–O distances are located in the range of 2.430–2.637 and 2.293–2.569 Å, respectively. The Ag_36_ skeleton consists of 8 trigons and 18 tetragons, which are edge-fused together to form the Ag_36_ shell. The μ_4_-^*i*^PrS^−^ exclusively bonded to tetragons, whereas *p*-TOS^−^ coordinated to both trigons on Ag_9_S_3_ caps and partial tetragons on Ag_18_S_12_ barrel. In **SD/Ag12**, Ag⋯Ag distances between 2.926 and 2.354 Å; are observed. The dimensions of the Ag_36_ shell account for ~1.4 nm in length and 0.9 nm in width. The most fascinating feature of **SD/Ag12** is the inner Mo_5_O_18_^6−^ core, which is built from two octahedral MoO_6_, two square-pyramidal MoO_5_ and one tetrahedral MoO_4_ units (Fig. 5d). The 36 silver atoms aggregated around the surface of Mo_5_O_18_^6−^ forming the core–shell structure. Among 36 silver atoms, 25 are coordinated to both terminal and bridge O atoms (Fig. 5e). Such Mo_5_O_18_^6−^ may be transient and can only be stabilized in the void of silver cluster through the rich Ag–O bonding.

From the above structural analyses, we found that (i) the same POM precursor can transform to different POMs in different solvents (MeOH vs MeCN); (ii) Mo-based anions with rich geometries and compositions templated diverse silver clusters; (iii) DMF plays an important role in the reductive formation of subvalent silver kernel; (iv) multiple simple and small anion templates (MoO_4_^2−^) can also induce the formation of large silver clusters through special arrangement of the anions. The multiple roles of solvents promise rational access to more complex and diverse silver clusters with special geometries or symmetries.

### Electrospray ionization mass spectrometry

**Fig. 6:**
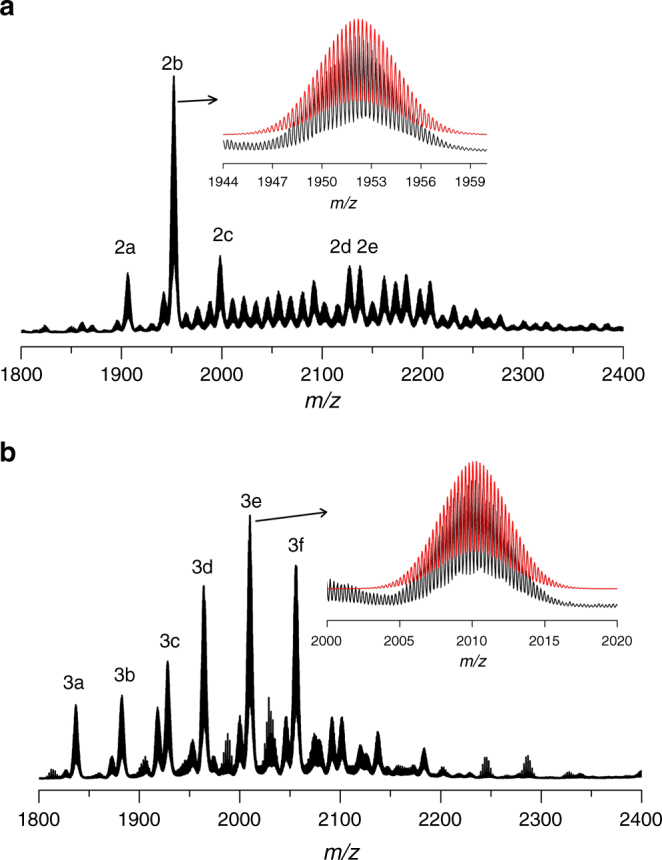
The solution behaviours of **SD/Ag8** and **SD/Ag9**. Positive-mode ESI-MS of **SD/Ag8** (**a**) and **SD/Ag9** (**b**) dissolved in acetonitrile. Insets: Comparison of the experimental (black spectrum) and simulated (superimposed red spectrum) isotopic envelopes for **2b** and **3e**

To detect the stability of the cluster, the ESI-MS of **SD/Ag8**, **SD/Ag9** and **SD/Ag11** dissolved in acetonitrile were performed in positive-ion mode. As depicted in Fig. 6a, b, **SD/Ag8** displays five identifiable species (**2a**–**2e**), whereas **SD/Ag9** shows six species (**3a**–**3f**) with the charge state of +4 in the range of *m/z* *=* 1800–2400. After carefully analyzing these peaks in the ESI-MS of **SD/Ag8** and **SD/Ag9**, we did not find any parent [Ag_6_@(MoO_4_)_7_@Ag_56_] species, which means neither **SD/Ag8** nor **SD/Ag9** are stable in acetonitrile, however, fragments with the [Ag_6_@(MoO_4_)_7_] core were unambiguously detected. The dominating species (**2b** and **3e**), centred at *m/z* *=* 1952.2731 and 2010.2228, can be assigned to [Ag_6_@(MoO_4_)_7_@Ag_35_(^*i*^PrS)_11_(*p*-TOS)_6_Cl(OH)_3_(CH_3_CN)_4_(H_2_O)_9_]^4+^ (Calc. *m/z* *=* 1952.2102) and [Ag_6_@(MoO_4_)_7_@Ag_36_(^*i*^PrS)_13_(CF_3_SO_3_)_9_(H_2_O)_4_]^4+^ (Calc. *m/z* *=* 2010.2902), respectively. The other species in the ESI-MS of **SD/Ag8** and **SD/Ag9** were identified based on the experimental and theoretical isotope distributions and presented in the Supplementary Figures 6, 7 and Supplementary Tables 3, 4. All species **2a**–**2e** and **3a**–**3f** have cores of [Ag_6_@(MoO_4_)_7_@Ag_*n*_] (*n*_**2a**–**2e**_ = 32 – 36 and *n*_**3a**–**3f**_ = 32 – 37), which indicates that the innermost [Ag_6_@(MoO_4_)_7_] core in **SD/Ag8** and **SD/Ag9** is stable in solution, despite the absence of the complete Ag_56_ parent shell. Considering the charge state of the assigned species, the subvalent nature of Ag_6_^4+^ was further justified by the ESI-MS results. With respect to outer ligand shell, the Δ*m/z* between **3a** and **3b** is 45.48, which is similar to the Δ*m/z* (45.98) between **3b** and **3c**, and the mass difference corresponds to one Ag^*i*^PrS, indicating the coordination–dissociation equilibrium with losing or adding Ag^*i*^PrS one by one in solution. Based on the above results, the presence of seven MoO_4_^2−^ anions enwrapping an Ag_6_ kernel should be precursor species, which is then closed by the outer Ag_56_ wheel at a second step in the growth process. The overall hierarchical cluster-in-cluster growth process was thus established for Ag_62_ cluster family, which was further justified by analyzing the reaction mixture by ESI-MS before crystallization (Supplementary Figure 4 and Supplementary Table 2).

**Fig. 7:**
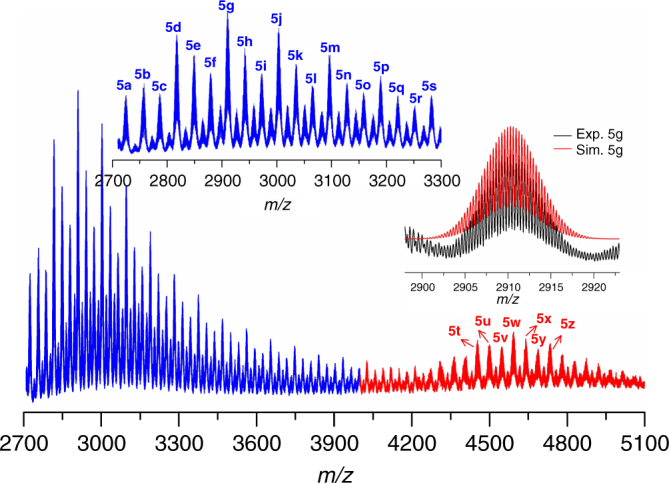
Positive-ion ESI-MS of **SD/Ag11** dissolved in acetonitrile. Insets: The enlarged MS of triply-charged species (blue line) and comparison of the experimental (black spectrum) and simulated (superimposed red spectrum) isotopic envelopes for **5g**

As shown in Fig. 7, the species found in ESI-MS of **SD/Ag11** dissolved in acetonitrile could be divided into two groups based on their charge states, +3 (**5a**–**5s**) and +2 (**5t**–**5z**), respectively, in the range of *m/z* = 2700–5100. The most abundant peak in the triply-charged species (see the inset of Fig. 7) located at *m/z* *=* 2910.2894 (**5** **g**), which can be assigned to [Mo_7_O_24_@Ag_41_(^*i*^PrS)_18_(*p*-TOS)_9_Cl_5_(CH_3_CN)_4_(H_2_O)]^3+^ (Calc. *m/z* *=* 2910.2880) and the most abundant peak in doubly-charged species centred at *m/z* *=* 4592.8767 (**5w**), which can be attributed to [Mo_7_O_24_@Ag_41_(^*i*^PrS)_10_(*p*-TOS)_15_Cl_6_(OH)_2_(CH_3_CN)_3_(H_2_O)]^2+^ (Calc. *m/z* *=* 4592.8338). The other 24 species were also identified and are shown in the Supplementary Figure 8 and Supplementary Table 5. Compared to the crystallographic result of **SD/Ag11**, we discovered that all species have the identical [Mo_7_O_24_@Ag_41_] skeleton, which indicates the core of **SD/Ag11** is intact in acetonitrile.

### UV–Vis absorption spectra and luminescence properties

**Fig. 8:**
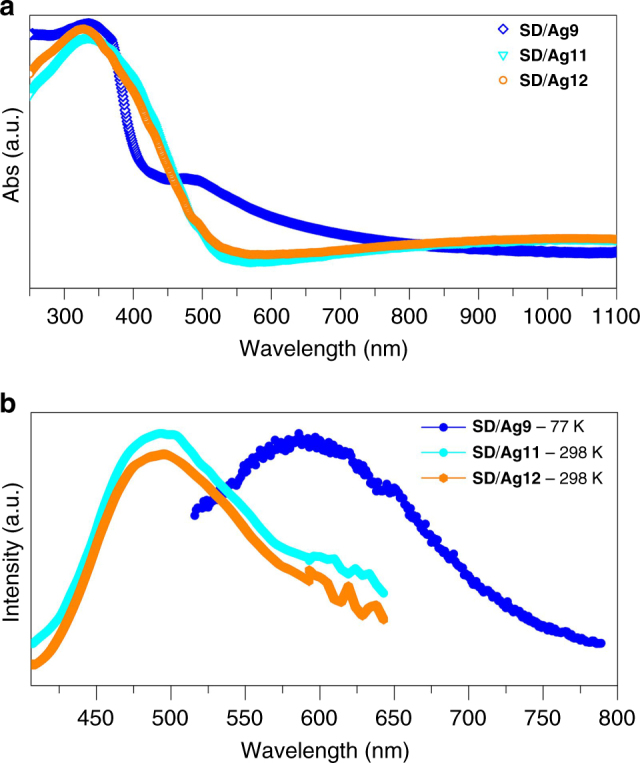
The spectral properties of **SD/Ag9**, **SD/Ag11** and **SD/Ag12**. The solid-state UV–Vis (**a**) and emission spectra (**b**) of the clusters **SD/Ag9**, **SD/Ag11** and **SD/Ag12**

The solid-state optical diffuse reflectance spectra of **SD/Ag9**, **SD/Ag11** and **SD/Ag12** were investigated at room temperature. As shown in Fig. 8a, compound **SD/Ag9** exhibits one main peak centred at 338 nm and weak shoulder peak at 483 nm, which should be assigned to the *n* → π^*^ transition of ^*i*^PrS^−^and ligand-to-metal charge transfer (LMCT) transition, respectively. However, compounds **SD/Ag11** and **SD/Ag12** show only one peak at 335 and 328 nm, respectively, both belongs to the *n* → π^*^ transition of ^*i*^PrS^−^.

Furthermore, the photoluminescence properties of **SD/Ag9**, **SD/Ag11** and **SD/Ag12** were also studied in the solid state. As shown in Fig. 8b, compound **SD/Ag9** is emission silent at room temperature, however, it emits yellow light at 77 K with a maximum at 587 nm (*λ*_ex_ = 400 nm), which is probably due to a ligand-to-metal charge transfer (LMCT, charge transfer from the S 3p to Ag 5s orbital) transition disturbed by Ag⋯Ag interactions^65^. This temperature-sensitive emission behaviours should be related to the variations of molecule rigidity and argentophilicity under different temperatures and have been previously observed in other silver-thiolate clusters such as [(CO_3_)@Ag_20_(SBu^*t*^)_10_(DMF)_6_(NO_3_)_8_]^66^ and [S@Ag_56_S_12_(SBu^*t*^)_20_](CH_3_COO)_10_^67^. Compounds **SD/Ag11** and **SD/Ag12** emit green light at room temperature with a similar maximum emission peak at *ca*. 493 nm upon the excitation at 330 nm, which should be attributed to the LMCT transition, or mixed with Ag⋯Ag interactions.

## Discussion

In summary, we discovered a family of sevenfold symmetric silver nanoclusters featuring the anisotropic wheel-like geometry encapsulating an octahedral nanofragment of metallic silver inside. The formation of such nanoclusters was realized by solvent modulation, which not only dictates the formation of octahedral Ag_6_^4+^ kernel, but also influence the in-situ-generated Mo-based templates. Different anions, *p*-TOS^−^, CF_3_SO_3_^−^ and NO_3_^−^, were involved in four wheel-like silver-thiolate clusters but without breaking the basic wheel-like backbones. The overall Ag_62_ nanocluster features a hierarchical cluster-in-cluster structure comprising an octahedral Ag_6_^4+^ kernel in the centre with up to seven MoO_4_^2−^ as templates around it, supporting the outermost Ag_56_ shell. The formation of an inner octahedral Ag_6_^4+^ kernel is closely dependent on the DMF as reducing agent. The unique sevenfold odd symmetry should be dictated by the prearranged seven MoO_4_^2−^ templates around the Ag_6_^4+^ kernel. In the absence of DMF, two different high-nuclear silver-thiolate clusters were identified without the Ag_6_^4+^ kernel trapped in the inner space but instead two POM templates were found and in situ generated from the same starting POM precursor. The present study launches a more rational approach to construct high-nuclearity silver clusters in which both the formation of octahedral Ag_6_^4+^ kernel and in situ generation of various Mo-based anion templates is controlled by the solvents.

## Methods

### Synthesis of the (Ag^*i*^PrS)_*n*_

The precursor of (Ag^*i*^PrS)_*n*_ was prepared by the following reported procedure^20^. The solution of AgNO_3_ (30 mmol, 5.1 g) in 75 mL acetonitrile was mixed with 100 mL ethanol containing ^*i*^PrSH (30 mmol, 2.8 mL) and 5 mL Et_3_N under stirring for 3 h in the dark at room temperature, then the yellow powder of (AgS^*i*^Pr)_*n*_ was isolated by filtration and washed with 10 mL ethanol and 20 mL ether, then dried in the ambient environment (yield: 97%).

### Synthesis of SD/Ag7 and SD/Ag8

*p*-TOSAg (0.1 mmol, 27.9 mg) and (Ag^*i*^PrS)_*n*_ (0.05 mmol, 9.2 mg) together with [(*n*-C_4_H_9_)_4_N]_4_[α-Mo_8_O_26_] (0.0002 mmol, 4.2 mg) were dissolved in a mixed solvent of methanol: *N,N*′-dimethylformamide (5 mL, v/v = 4/1). The reaction mixture was sealed and heated at 65 °C for 2000 min, and then cooled to room temperature for 800 min. Then, the brown solution was filtered and the filtrate left to evaporate slowly for 2 weeks in the dark at room temperature. Brown block crystals of **SD/Ag7** and diamond crystals of **SD/Ag8** were crystallized in the yields of 10% and 13%, respectively.

Elemental analyses calc. (found) for **SD/Ag7**: C_194_H_322_Ag_62_Mo_9_N_4_O_82_S_42_: C, 18.03 (18.12); H, 2.51 (2.59); N, 0.43 (0.39)%. Selected IR peaks (cm^−1^): 2910 (w), 1441 (w), 1374 (w), 1248 (w), 1144 (m), 1115 (m), 1003 (m), 781 (m), 677 (s), 595 (s), 555 (s).

Elemental analyses calc. (found) for **SD/Ag8**: C_191_H_315_Ag_62_Mo_9_N_3_O_81_S_42_: C, 17.86 (17.79); H, 2.47 (2.51); N, 0.33 (0.31)%. Selected IR peaks (cm^−1^): 2934 (w), 1621 (w), 1486 (w), 1451 (w), 1373 (w), 1245 (w), 1153 (s), 1117 (s), 1004 (s), 784 (s), 677 (s), 600 (w), 564 (s).

### Synthesis of SD/Ag9

The synthetic condition was similar to that described for **SD/Ag7** and **SD/Ag8**, except that the *p*-TOSAg was substituted by CF_3_SO_3_Ag (0.1 mmol, 25.6 mg), brown block crystals of **SD/Ag9** were crystallized in a yield of 35% after 3 days.

Elemental analyses calc. (found) for **SD/Ag9**: C_110_H_224_Ag_62_F_42_Mo_9_N_4_O_82_S_42_: C, 17.86 (17.90); H, 2.47 (2.55); N, 0.32 (0.29)%. Selected IR peaks (cm^−1^): 2952 (w), 1643 (w), 1448 (w), 1373 (w), 1224 (m), 1167 (m), 1011 (m), 784 (m), 635 (s), 511 (m).

### Synthesis of SD/Ag10

The synthetic condition was similar to that described for **SD/Ag7** and **SD/Ag8**, except that the *p*-TOSAg was replaced by AgNO_3_ (0.1 mmol, 17 mg), brown crystals of compound **SD/Ag10** were crystallized in a yield of 20% after 3 weeks.

Elemental analyses calc. (found) for **SD/Ag10**: C_84_H_196_Ag_62_Mo_9_N_14_O_78_S_28_: C, 9.09 (9.13); H, 1.78 (1.69); N, 1.77 (1.79)%. Selected IR peaks (cm^−1^): 2948 (w), 1650 (w), 1526 (w), 1451 (w), 1387 (m), 1267 (m), 1146 (m), 1047 (m), 776 (s), 600 (m).

### Synthesis of SD/Ag11

The synthetic condition was similar to that described for **SD/Ag7** and **SD/Ag8** but using MeOH (5 mL) instead, yellow rod crystals of **SD/Ag11** were isolated by filtration, washed with EtOH and dried in air (yield: 35%).

Elemental analyses calc. (found) for **SD/Ag11**: C_177_H_277_Ag_41_Mo_7_O_80_S_35_: C, 21.47 (21.51); H, 2.82 (2.79) %. Selected IR peaks (cm^−1^): 2927 (w), 1600 (w), 1500 (w), 1444 (w), 1380 (w), 1249 (w), 1110 (s), 1004 (s), 869 (m), 841 (m), 805 (m), 677 (s), 613 (m), 550 (s).

### Synthesis of SD/Ag12

The synthetic condition was similar to that described for **SD/Ag7** and **SD/Ag8** but using MeCN (5 mL) instead, yellow block crystals of compound **SD/Ag12** were crystallized in a yield of 20% after 10 days.

Elemental analyses calc. (found) for **SD/Ag12**: C_177.5_H_283_Ag_36_Mo_5_N_4_O_58.5_S_31.5_: C, 24.28 (24.19); H, 3.25 (3.28); N, 0.64 (0.61) %. Selected IR peaks (cm^−1^): 2934 (w), 1600 (w), 1495 (w), 1448 (w), 1382 (w), 1241 (w), 1117 (m), 1004 (s), 876 (w), 812 (m), 677 (s), 600 (m), 556 (s).

### Data availability

The X-ray crystallographic coordinates for structures reported in this article have been deposited at the Cambridge Crystallographic Data Centre, under deposition number CCDC: 1815301-1815304, 1815357 and 1815305 for SD/Ag7-SD/Ag12. These data can be obtained free of charge from the Cambridge Crystallographic Data Centre via www.ccdc.cam.ac.uk/data_request/cif.

## Electronic supplementary material

Supplementary Information

Peer Review File
