# Supplementary material for: Trapping an octahedral Ag6 kernel in a seven-fold symmetric Ag56 nanowheel
Source: Nat Commun. 2018 May 29;9:2094. doi: 10.1038/s41467-018-04499-9 (PMC5974400; doi:10.1038/s41467-018-04499-9)
Supplement: Supplementary file 1 — Supplementary Information [file 41467_2018_4499_MOESM1_ESM.pdf]

Supplementary Information

Trapping an Octahedral Ag_6 Kernel in a Seven-Fold Symmetric Ag_{56} Nanowheel

Zhi Wang,[1] Hai-Feng Su,[2] Mohamedally Kurmoo,[3] Chen-Ho Tung,[1] Di Sun[*,1] and Lan-Sun Zheng[2]

[1]Key Laboratory of the Colloid and Interface Chemistry, Ministry of Education, and School of Chemistry and Chemical Engineering, Shandong University, Jinan, 250100, P. R. China.

[2]State Key Laboratory for Physical Chemistry of Solid Surfaces and Department of Chemistry, College of Chemistry and Chemical Engineering, Xiamen University, Xiamen, 361005, P. R. China.

[3]Institut de Chimie de Strasbourg, Université de Strasbourg, CNRS-UMR 7177, 4 rue Blaise Pascal, 67008 Strasbourg Cedex, France.

*To whom correspondence should be addressed. E-mail: dsun@sdu.edu.cn

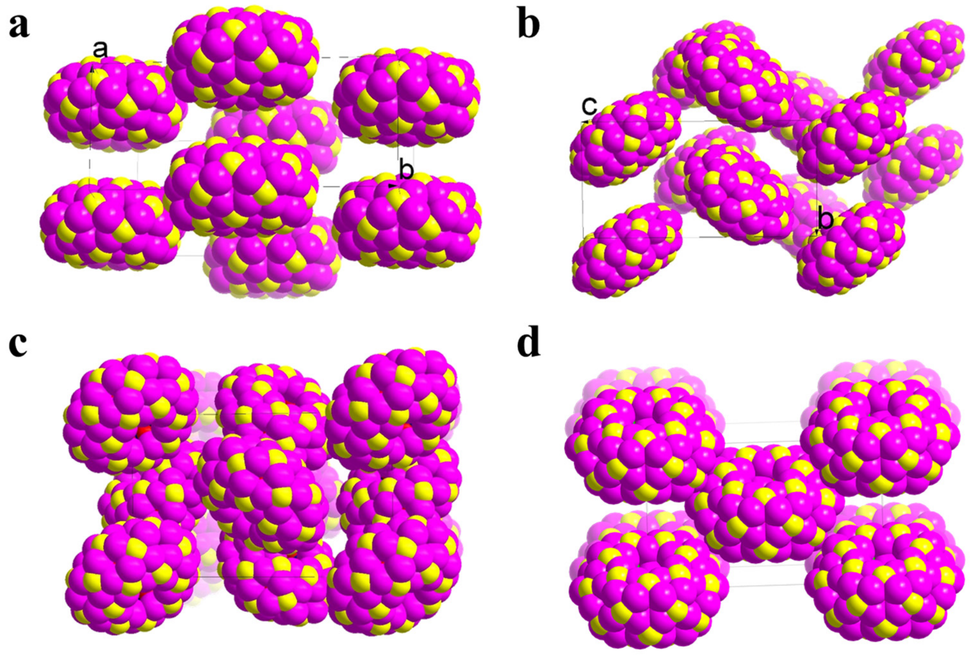

Supplementary Figure 1: Molecules packing in one unit cell for **SD/Ag7-SD/Ag10** (**a-d**).

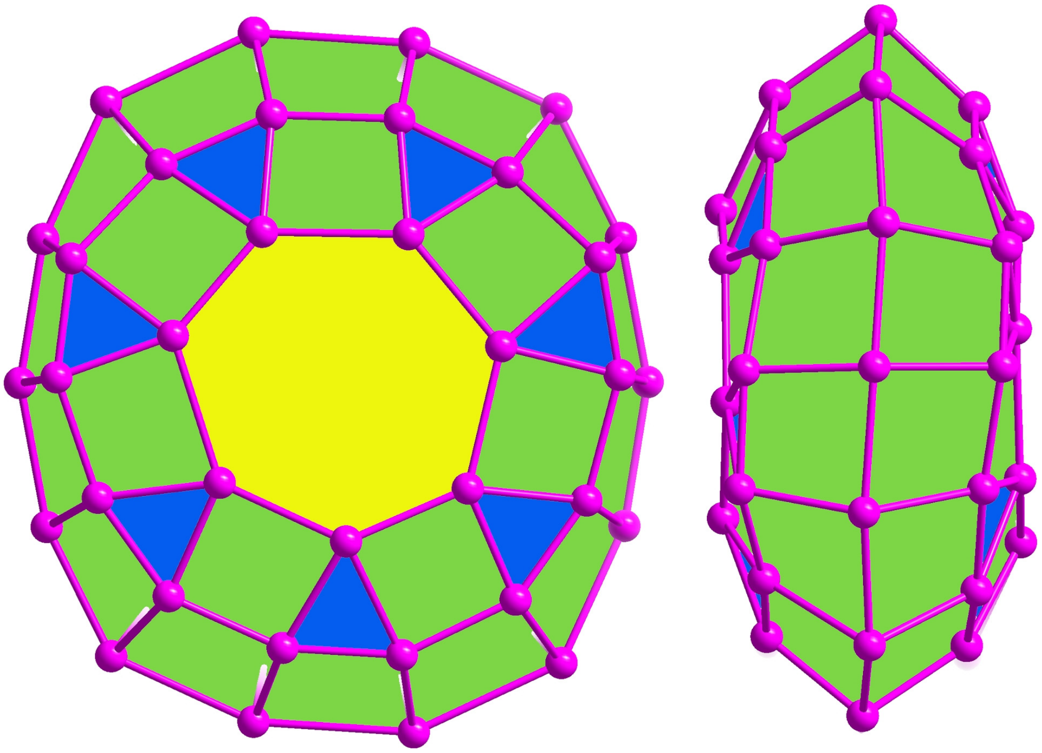

Supplementary Figure 2: The polygons on the surface of Ag_{56} wheel in **SD/Ag7.**

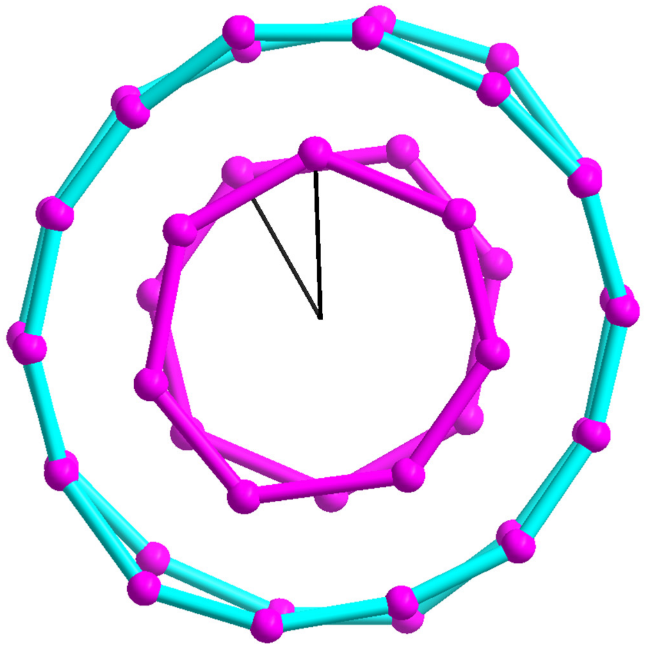

Supplementary Figure 3： The stacking modes of Ag_7 and Ag_{14} rings in **SD/Ag7.**

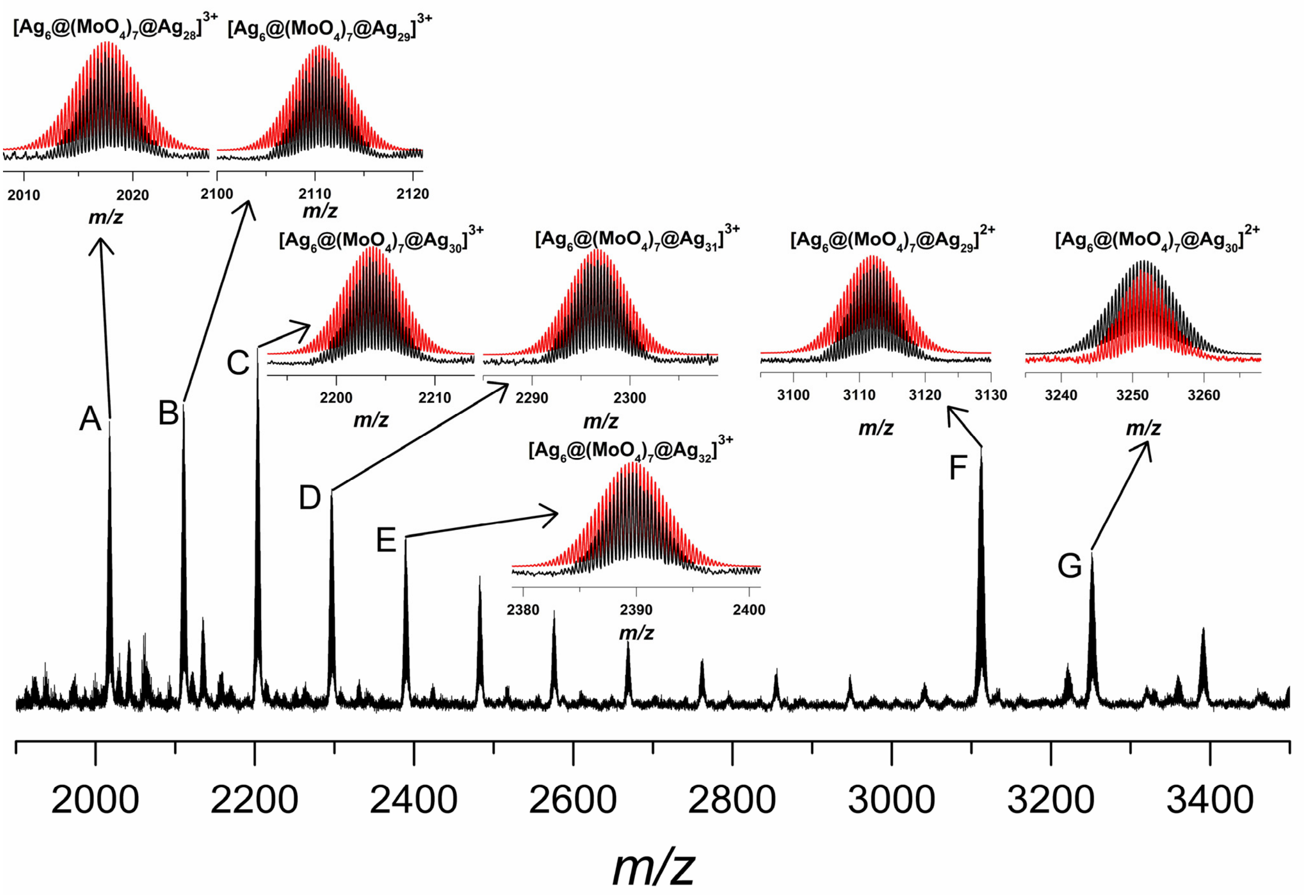

Supplementary Figure 4: The simulated and experimental isotopic distributions of species found in ESI-MS of an early-stage reaction mixture during the synthesis of **SD/Ag7.**

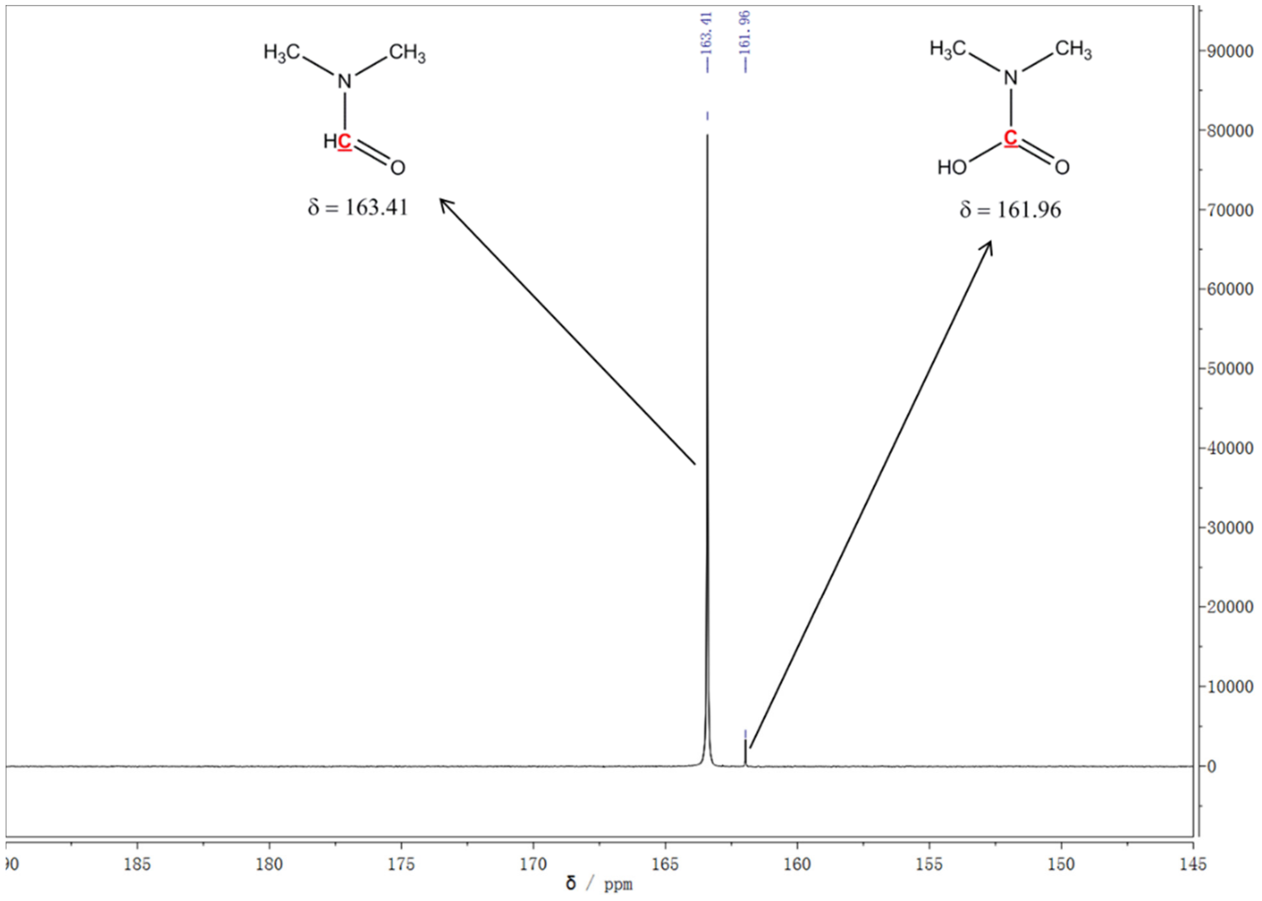

Supplementary Figure 5: ^{13}C NMR of HCl digested reaction mother solution for **SD/Ag7.**

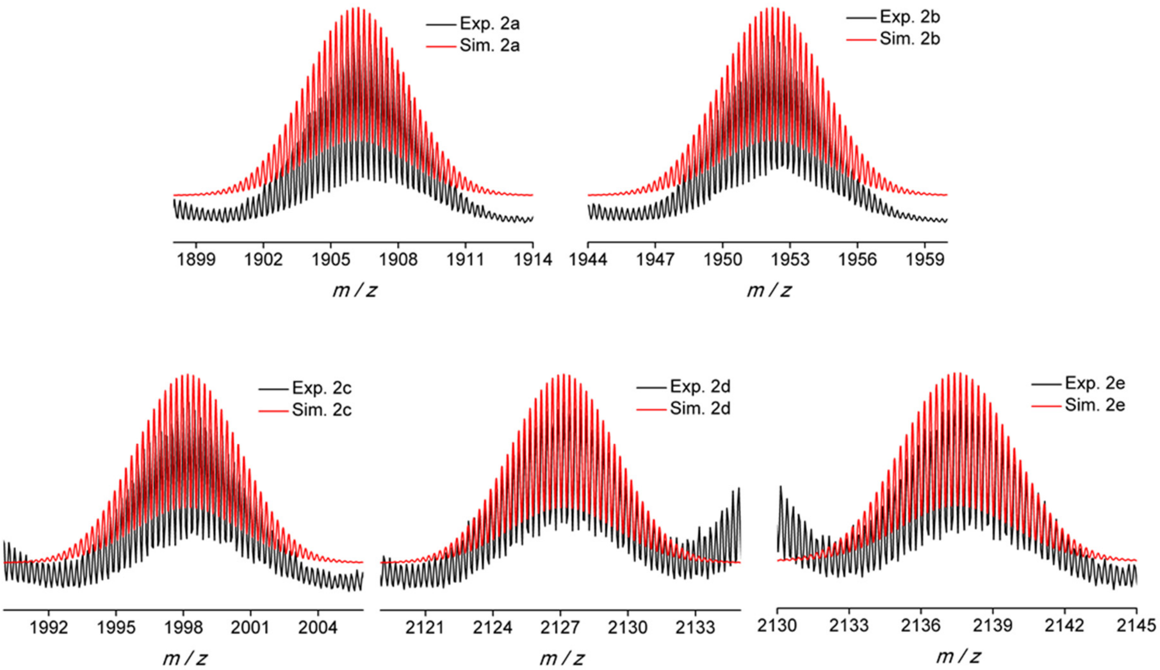

Supplementary Figure 6: The simulated and experimental isotopic distributions of species found in ESI-MS of **SD/Ag8** dissolved in CH_3CN.

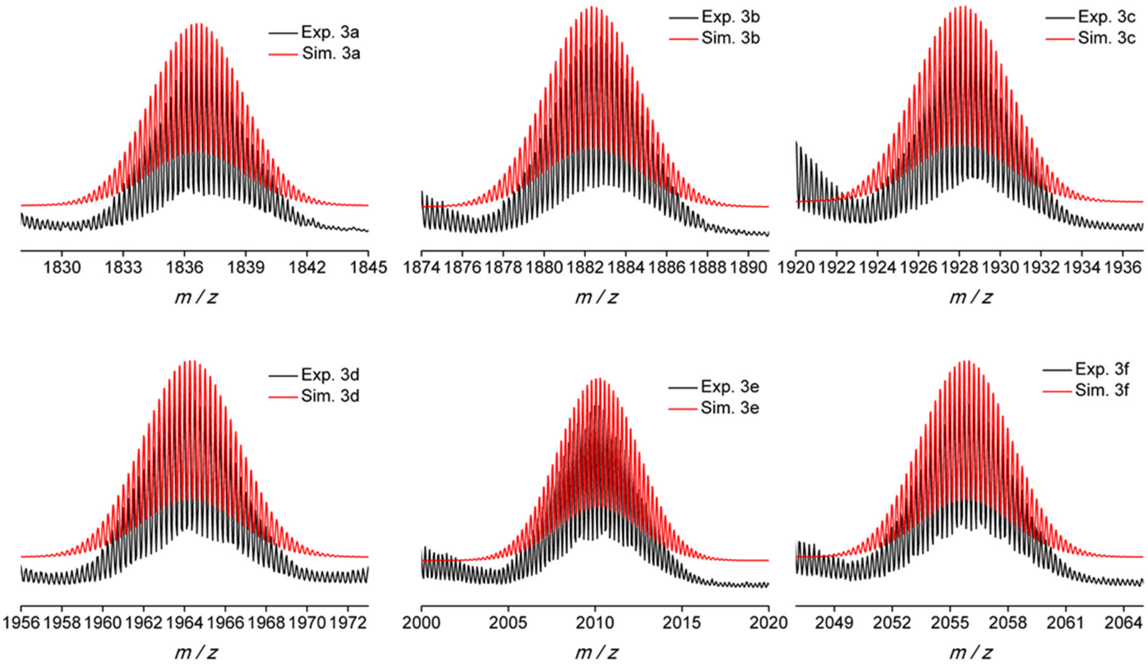

Supplementary Figure 7: The simulated and experimental isotopic distributions of species found in ESI-MS of **SD/Ag9** dissolved in CH_3CN.

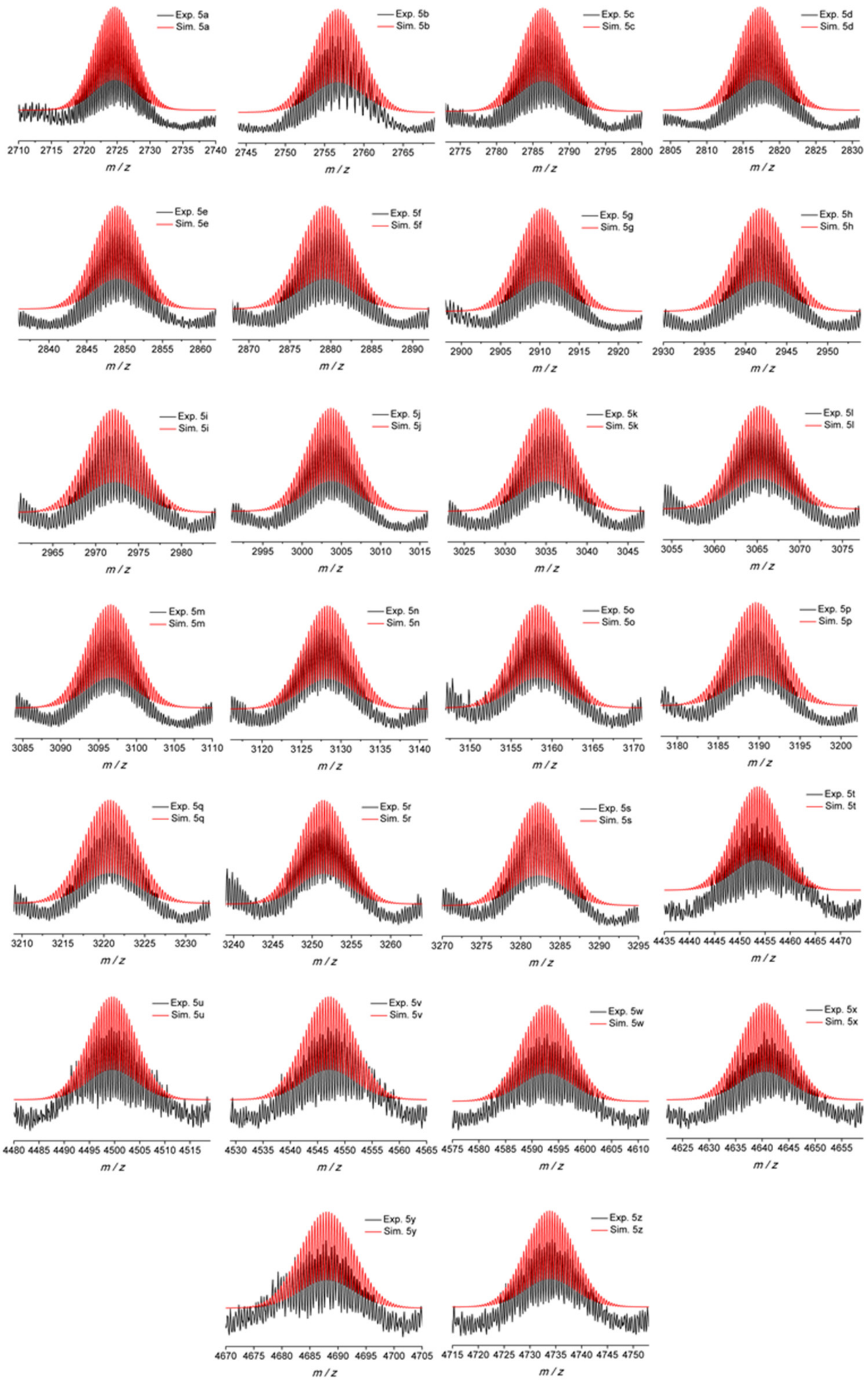

Supplementary Figure 8: The simulated and experimental isotopic distributions of species found in ESI-MS of **SD/Ag11** dissolved in CH_3CN.

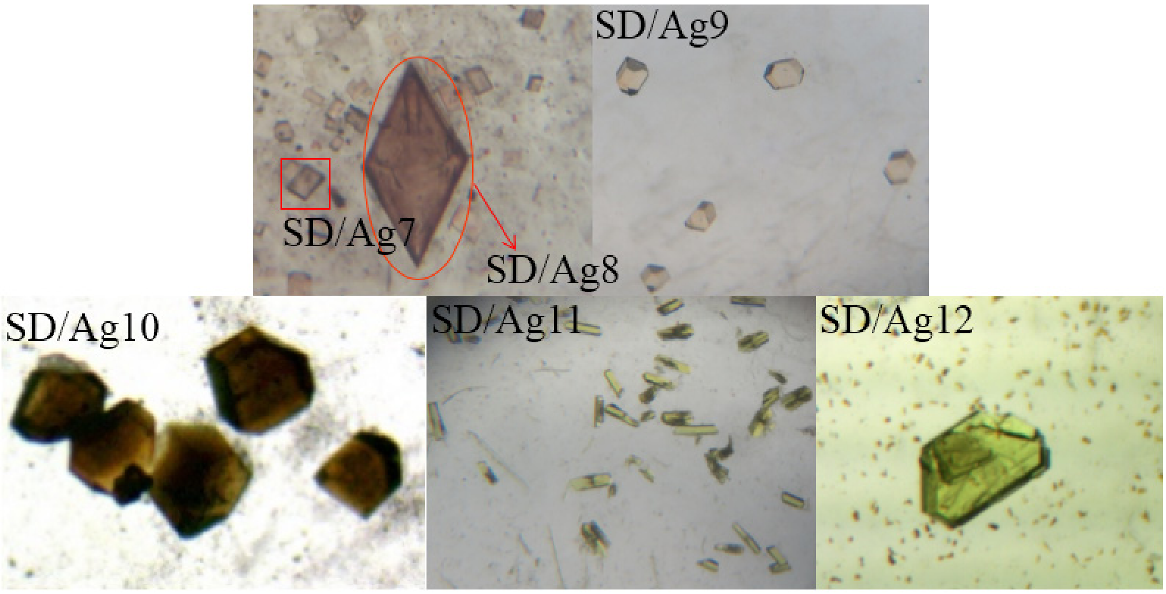

Supplementary Figure 9: Microscope photographs of crystals **SD/Ag7-SD/Ag12.**

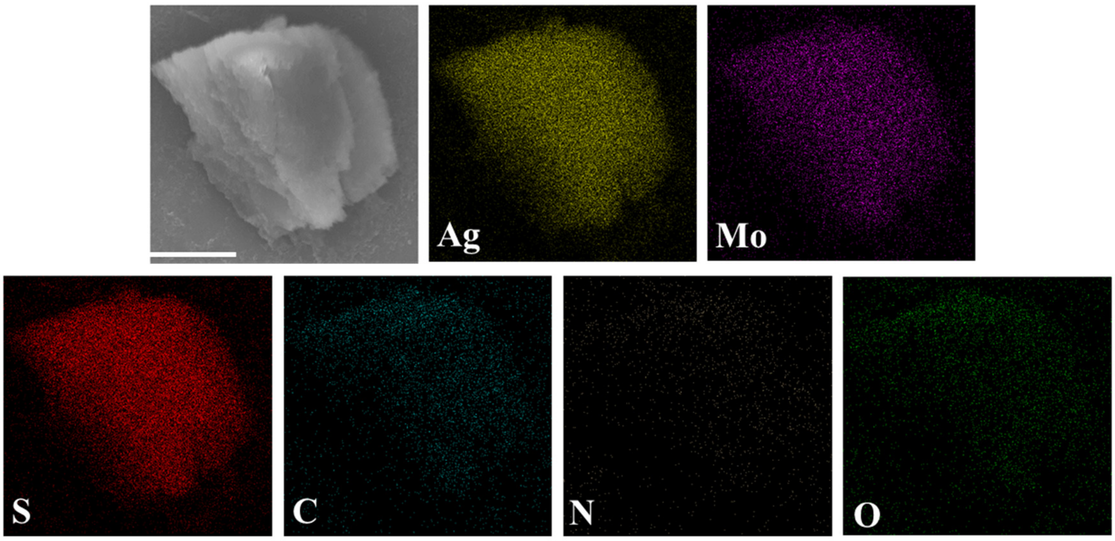

Supplementary Figure 10: The energy dispersive spectroscopy (EDS) mapping results on an SEM image of single particle of **SD/Ag7**. The scale bar is 5 μm.

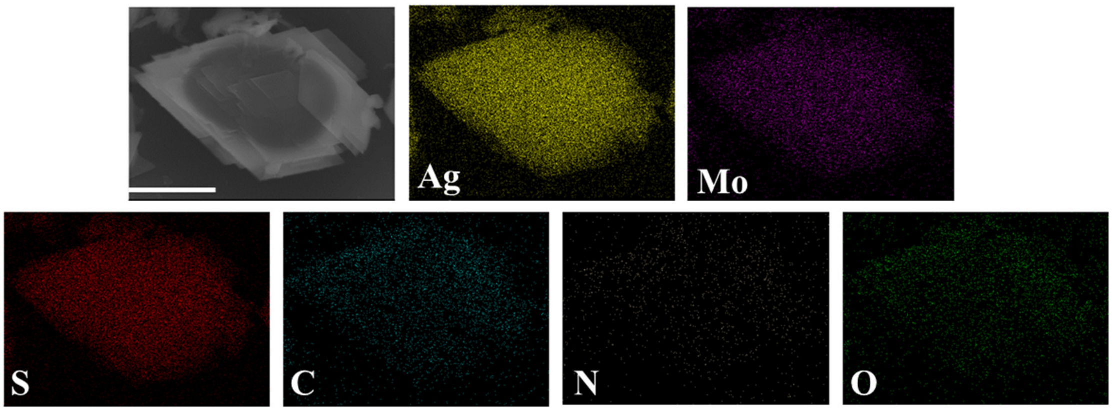

Supplementary Figure 11: The energy dispersive spectroscopy (EDS) mapping results on an SEM image of single particle of **SD/Ag8**. The scale bar is 5 μm.

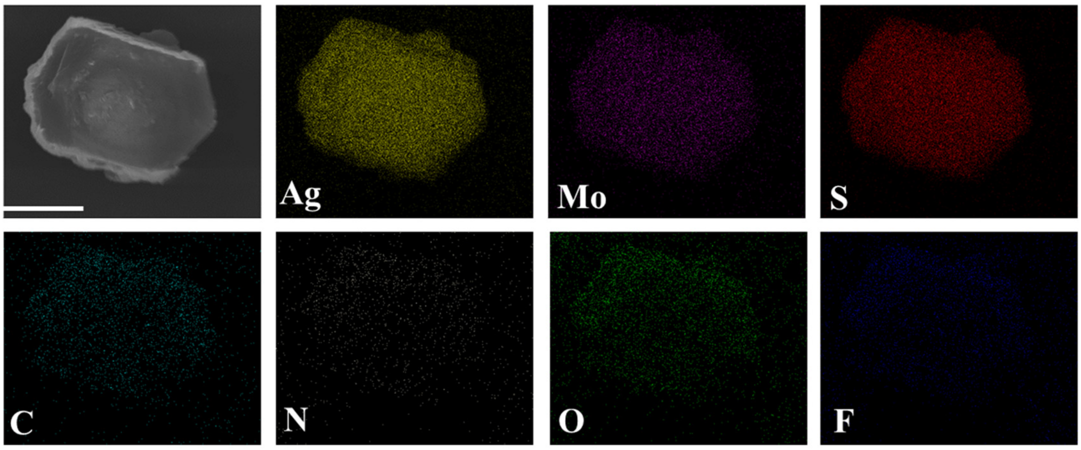

Supplementary Figure 12: The energy dispersive spectroscopy (EDS) mapping results on an SEM image of single particle of **SD/Ag9**. The scale bar is 5 μm.

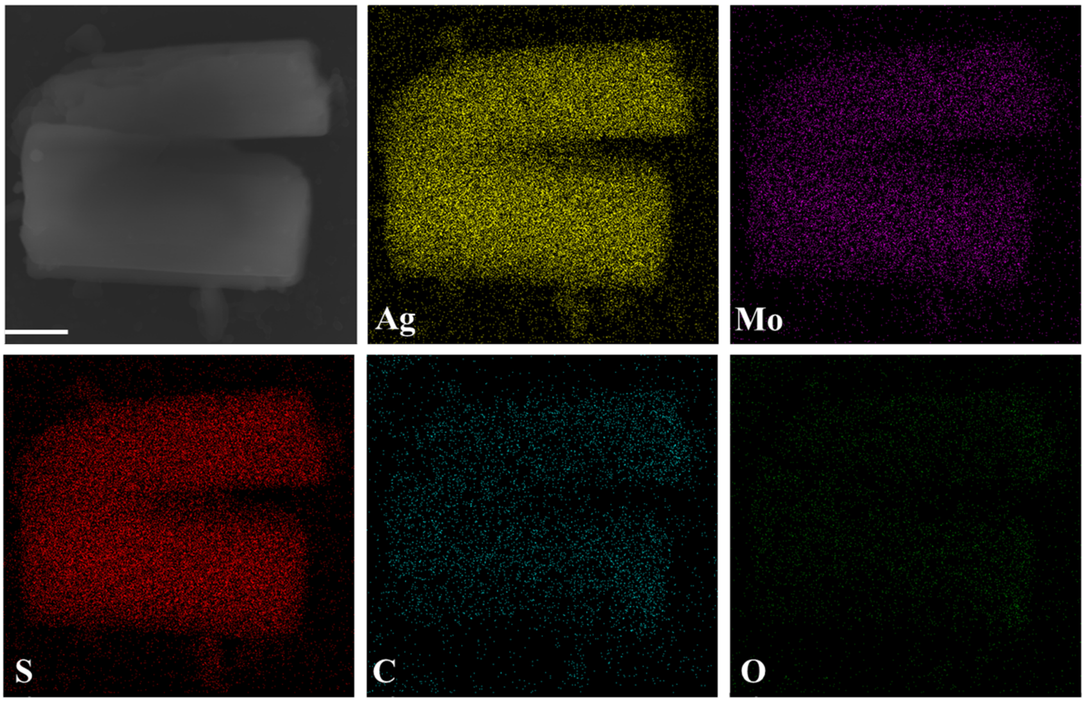

Supplementary Figure 13: The energy dispersive spectroscopy (EDS) mapping results on an SEM image of single particle of **SD/Ag11**. The scale bar is 2 µm.

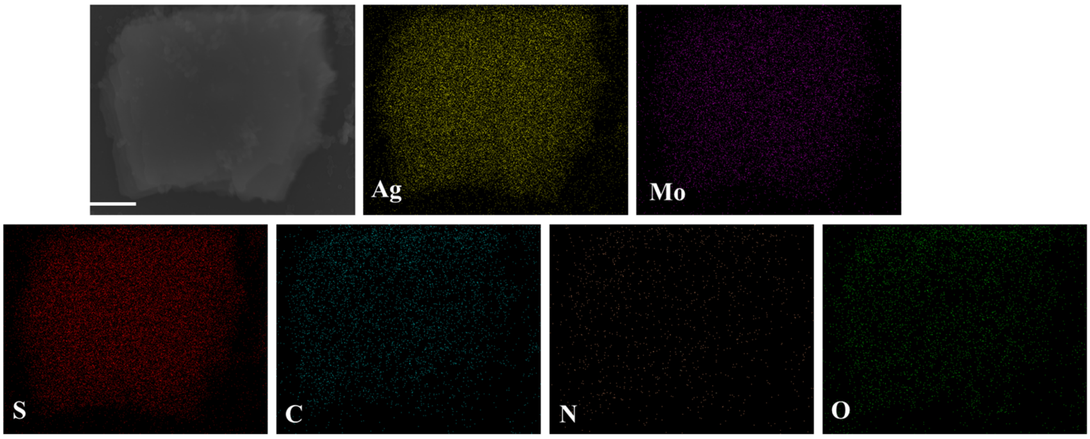

Supplementary Figure 14: The energy dispersive spectroscopy (EDS) mapping results on an SEM image of single particle of **SD/Ag12**. The scale bar is 2 μm.

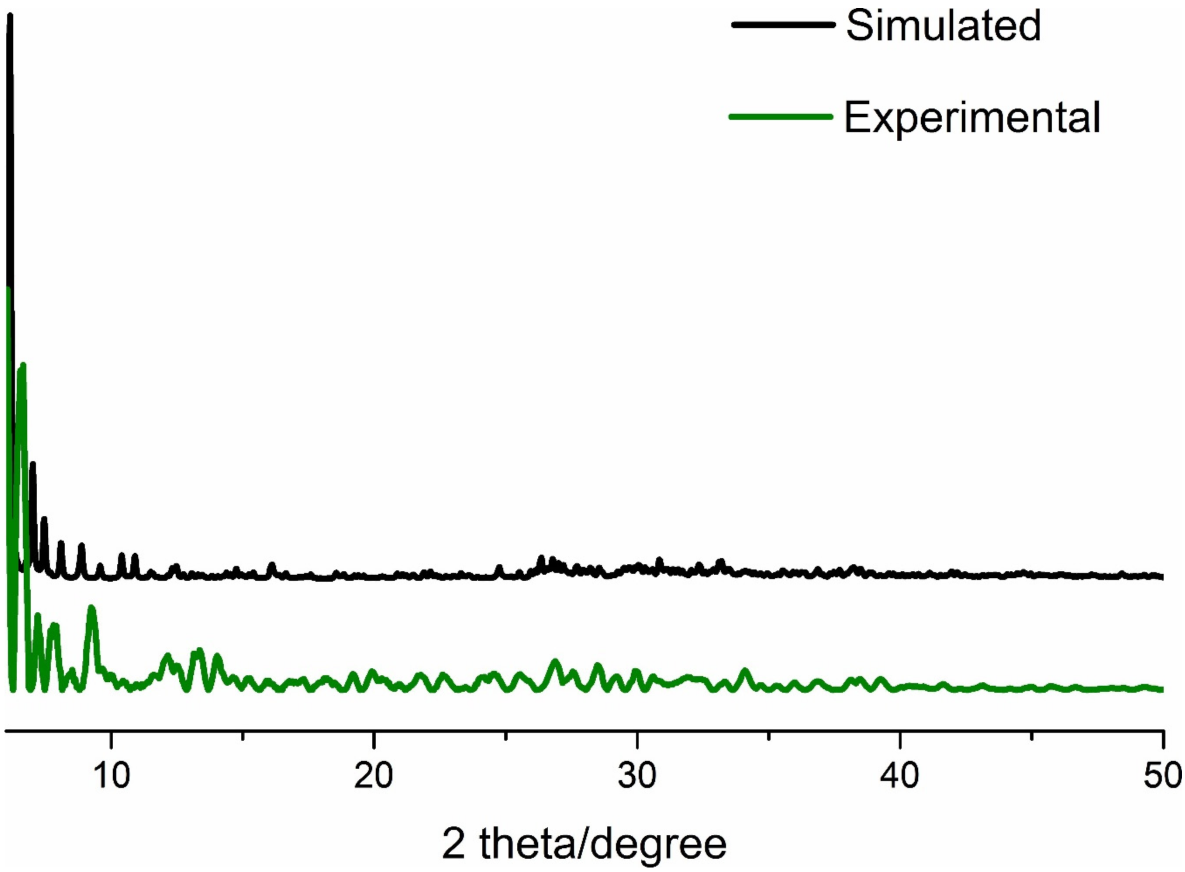

Supplementary Figure 15: Compared PXRD patterns of **SD/Ag7**.

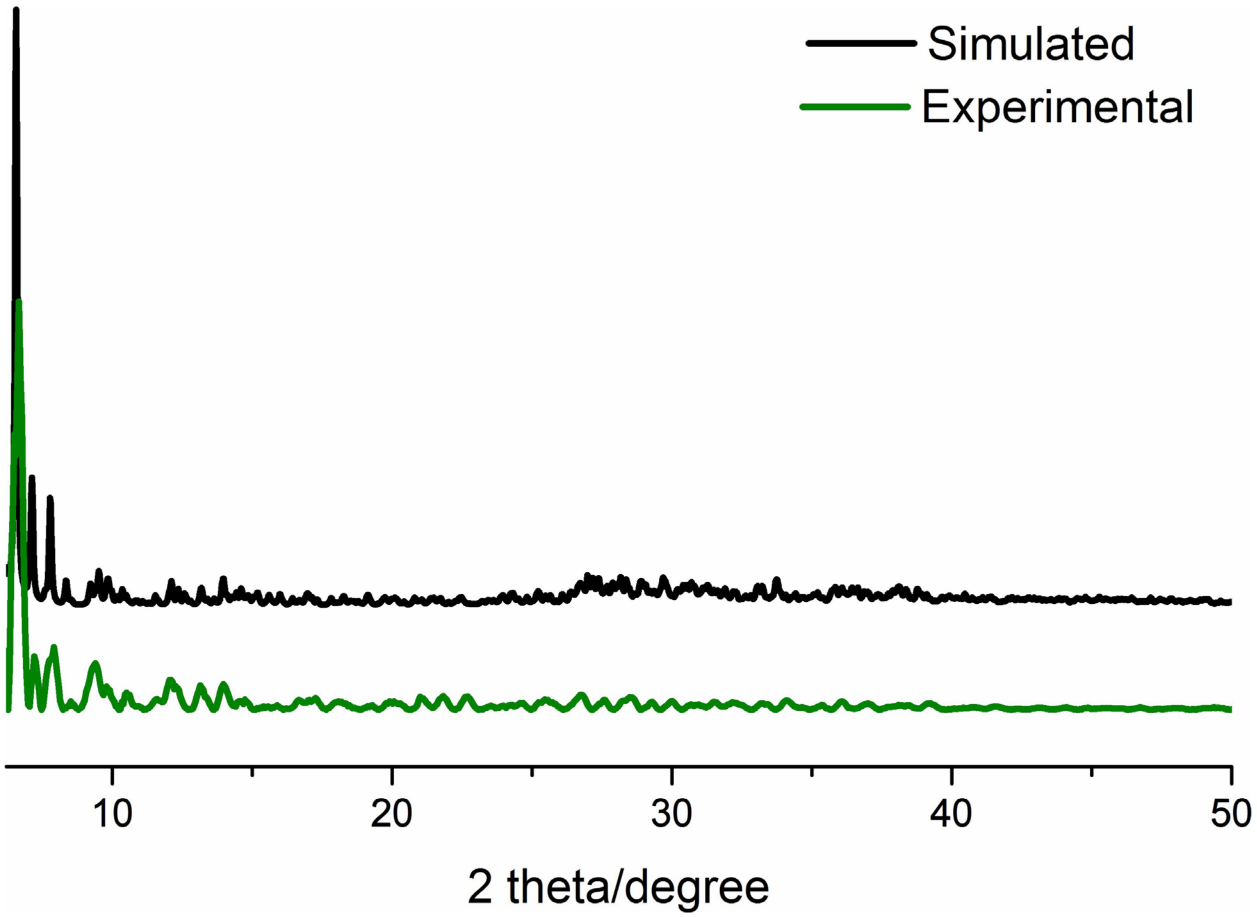

Supplementary Figure 16: Compared PXRD patterns of **SD/Ag8**.

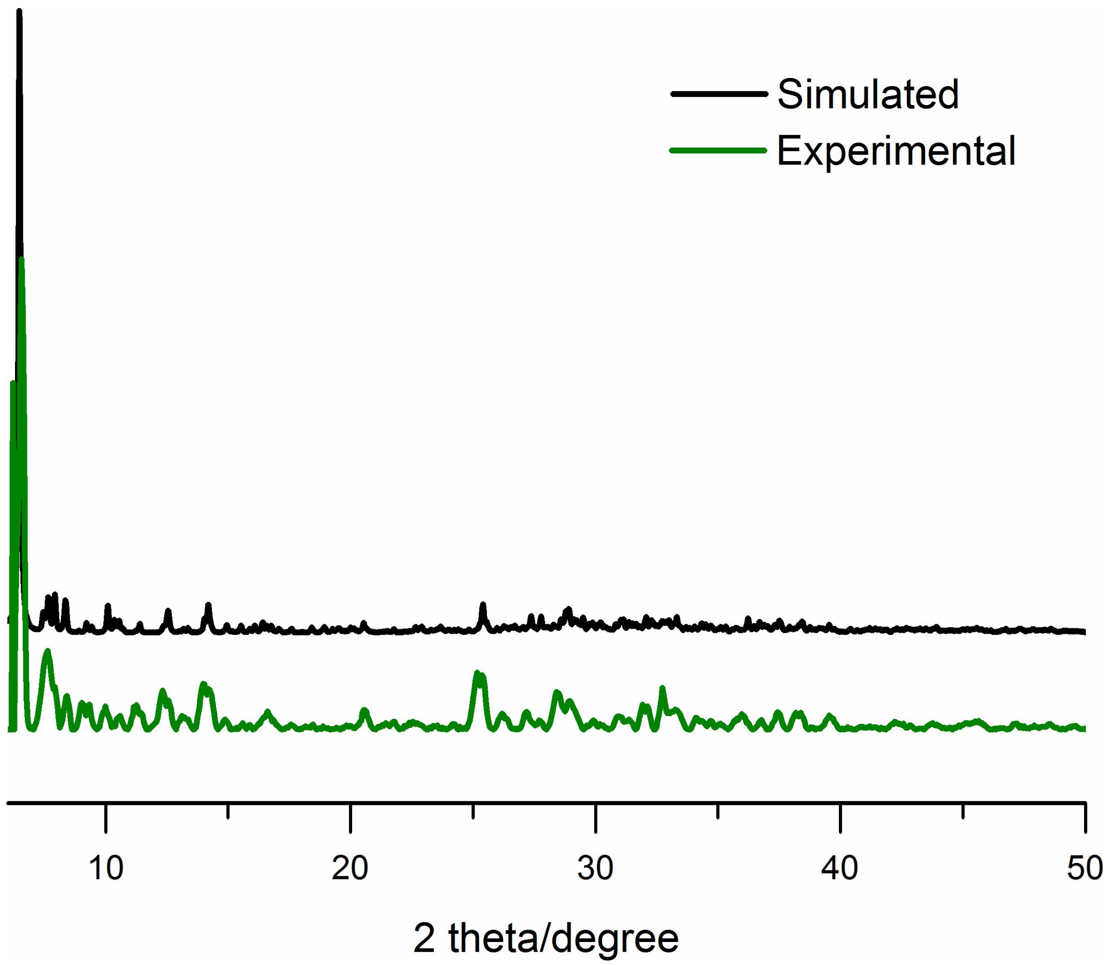

Supplementary Figure 17: Compared PXRD patterns of **SD/Ag9**.

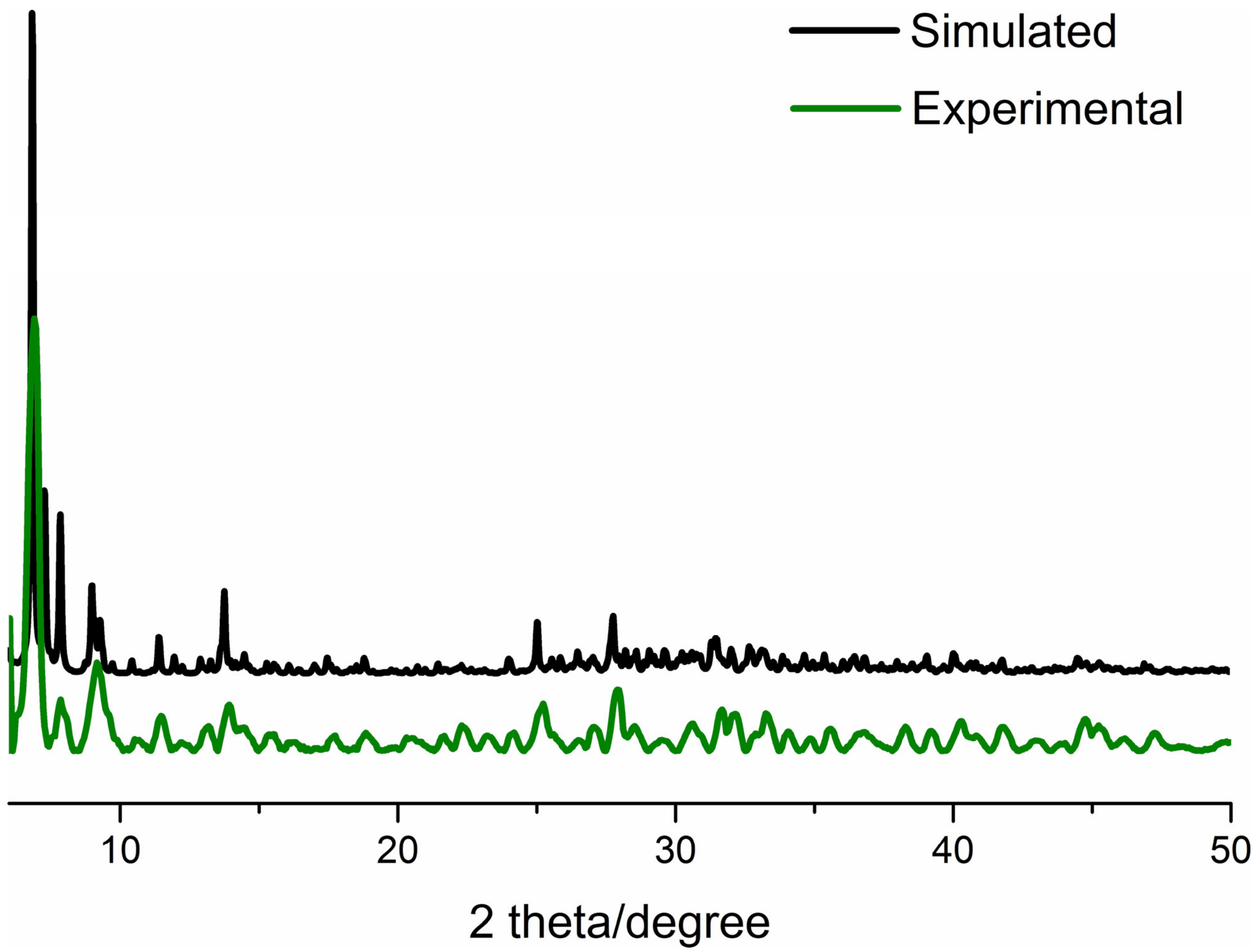

Supplementary Figure 18: Compared PXRD patterns of **SD/Ag10**.

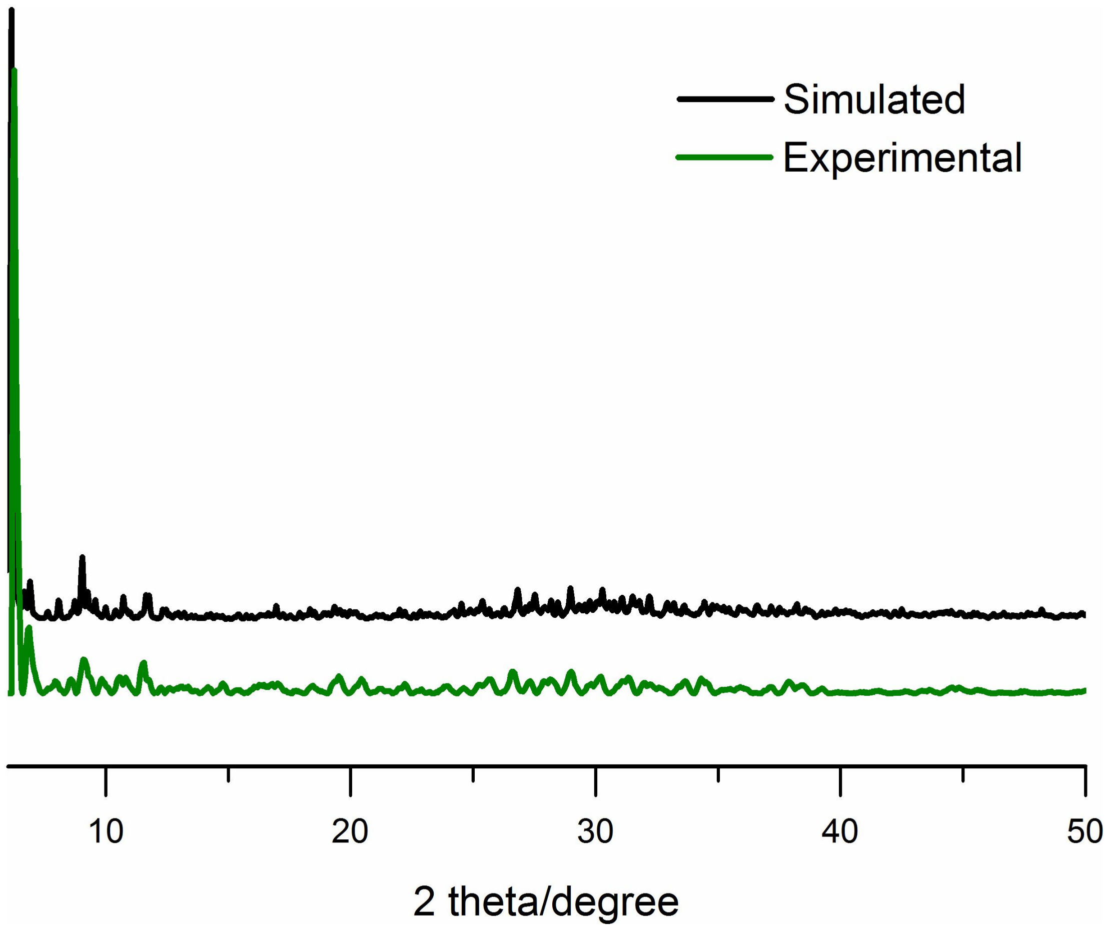

Supplementary Figure 19: Compared PXRD patterns of **SD/Ag11**.

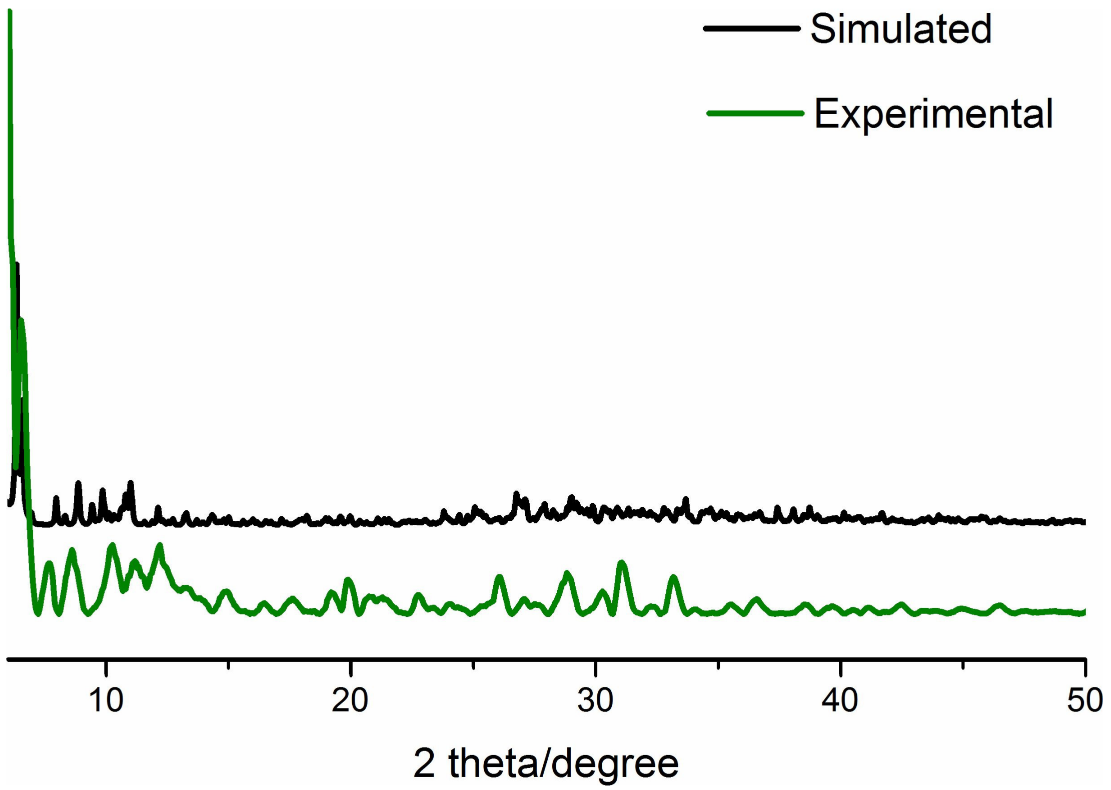

Supplementary Figure 20: Compared PXRD patterns of **SD/Ag12**.

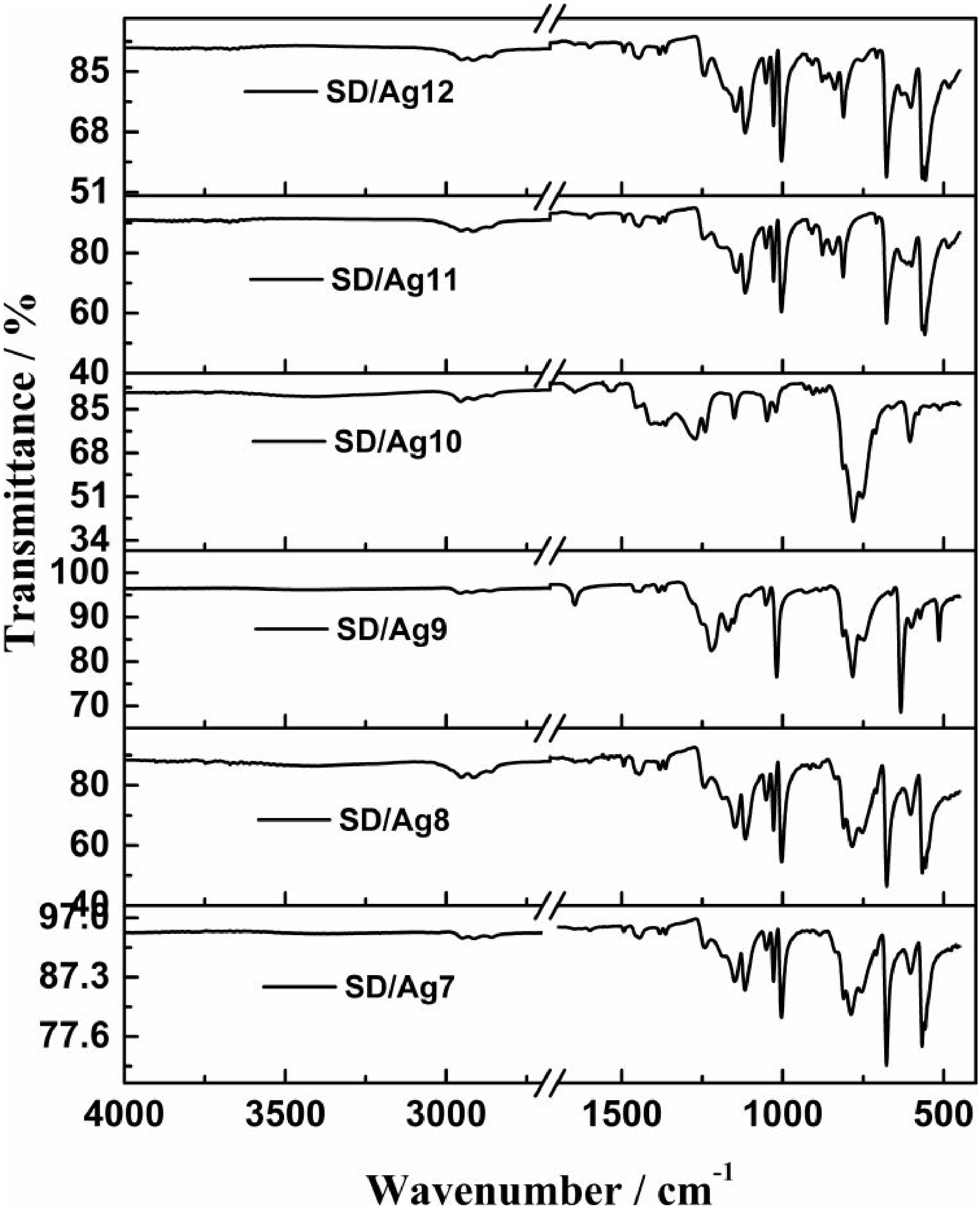

Supplementary Figure 21: The IR spectra of **SD/Ag7-SD/Ag12**.

Supplementary Table 1: Crystal Data Collection and Structure Refinement for **SD/Ag7-SD/Ag12.**

Compound	**SD/Ag7**	**SD/Ag8**	**SD/Ag9**	**SD/Ag10**	**SD/Ag11**	**SD/Ag12**
Empirical formula	$C_{194}H_{322}Ag_{62}Mo_9N_4O_{82}S_{42}$	$C_{191}H_{315}Ag_{62}Mo_9N_3O_{81}S_{42}$	$C_{110}H_{224}Ag_{62}F_{42}Mo_9N_4O_{82}S_{42}$	$C_{84}H_{196}Ag_{62}Mo_9N_{14}O_{78}S_{28}$	$C_{177}H_{277}Ag_{41}Mo_7O_{80}S_{35}$	$C_{177.5}H_{283}Ag_{36}Mo_5N_4O_{58.5}S_{31.5}$
X-ray diffractometer	Bruker APEX II	Bruker APEX II	Rigaku Oxford Diffraction XtaLAB Synergy	Rigaku Oxford Diffraction XtaLAB Synergy	Rigaku Oxford Diffraction XtaLAB Synergy	Bruker APEX II
Formula weight	12920.46	12847.36	12610.84	11099.62	9901.32	8782.17
Temperature/K	123(2)	100(2)	99.99(10)	100.00(10)	100.00(10)	100(2)
Crystal system	monoclinic	monoclinic	tetragonal	monoclinic	orthorhombic	triclinic
Space group	$P2_1$/c	*C*2/c	$P4_2$/ncm	*I*2/m	*P*ccn	*P*-1
a/Å	16.6512(2)	56.1786(9)	37.4406(5)	20.2995(8)	23.3347(3)	18.723(2)
b/Å	39.8911(6)	19.1872(3)	37.4406(5)	32.3201(19)	33.7522(4)	20.181(2)
c/Å	26.7451(4)	38.0666(5)	23.5067(5)	24.8471(18)	36.4441(4)	36.569(4)
α/°	90	90	90	90	90	84.7323(13)
β/°	102.7486(7)	118.0405(7)	90	109.744(6)	90	77.9249(13)
γ/°	90	90	90	90	90	62.8124(12)
Volume/Å^3	17327.1(4)	36215.8(10)	32951.5(10)	15343.4(16)	28703.2(6)	12018(2)
Z	2	4	4	2	4	2
ρ_{calc}g/cm^3	2.476	2.356	2.542	2.403	2.291	2.326
μ/mm^{-1}	4.0376	3.861	4.258	4.444	3.339	3.444
F(000)	12268.0	24376.0	23640.0	10324.0	19040.0	8083.0
Radiation	MoKα (λ = 0.71073)	MoKα (λ = 0.71073)	MoKα (λ = 0.71073)	MoKα (λ = 0.71073)	MoKα (λ = 0.71073)	MoKα (λ = 0.71073)
Reflections collected	96625	83937	148329	39356	223455	77670
Independent reflections	24824 [R_{int}= 0.0739, R_{sigma}= 0.0651]	25626 [R_{int}= 0.0462, R_{sigma}= 0.0499]	12054 [R_{int}= 0.0488, R_{sigma}= 0.0217]	11145 [R_{int}= 0.0275, R_{sigma}= 0.0264]	20422 [R_{int}= 0.0649, R_{sigma}= 0.0245]	26086 [R_{int}= 0.0316, R_{sigma}= 0.0471]
Data/parameters	24824/1969	25626/2006	12054/962	11145/630	20422/1590	26086/2682
Goodness-of-fit on F^2	1.043	1.081	1.093	1.041	1.118	1.030
Final R indexes [I>=2σ (I)]	R_1 = 0.0854, wR_2 = 0.2148	R_1 = 0.0873, wR_2 = 0.1969	R_1 = 0.0818, wR_2 = 0.2111	R_1 = 0.0892, wR_2 = 0.2417	R_1 = 0.0450, wR_2 = 0.0963	R_1 = 0.0707, wR_2 = 0.1690
Final R indexes [all data]	R_1 = 0.0998, wR_2 = 0.2248	R_1 = 0.1134, wR_2 = 0.2120	R_1 = 0.0982, wR_2 = 0.2274	R_1 = 0.1059, wR_2 = 0.2585	R_1 = 0.0588, wR_2 = 0.1072	R_1 = 0.0858, wR_2 = 0.1774
Largest diff. peak/hole / e Å^{-3}	2.49/-3.25	2.61/-2.20	3.00/-1.90	2.99/-1.94	1.90/-1.52	4.12/-1.65

Supplementary Table 2: The assigned formula of species found in ESI-MS of an early-stage reaction mixture during the synthesis of **SD/Ag7**.

**Peaks**	**Species**	**Exp. *m/z***	**Sim. *m/z***
**A**	$[Ag_6@(MoO_4)_7@Ag_{28}(^iPrS)_{13}(p\text{-}TOS)Cl(H_2O)(CH_3OH)_2]^{3+}$	2017.7840	2017.7851
**B**	$[Ag_6@(MoO_4)_7@Ag_{29}(^iPrS)_{13}(p\text{-}TOS)_2Cl(H_2O)(CH_3OH)_2]^{3+}$	2110.4260	2110.4240
**C**	$[Ag_6@(MoO_4)_7@Ag_{30}(^iPrS)_{13}(p\text{-}TOS)_3Cl(H_2O)(CH_3OH)_2]^{3+}$	2203.7278	2203.7295
**D**	$[Ag_6@(MoO_4)_7@Ag_{31}(^iPrS)_{13}(p\text{-}TOS)_4Cl(H_2O)(CH_3OH)_2]^{3+}$	2296.7001	2296.7017
**E**	$[Ag_6@(MoO_4)_7@Ag_{32}(^iPrS)_{13}(p\text{-}TOS)_5Cl(H_2O)(CH_3OH)_2]^{3+}$	2389.6739	2389.6708
**F**	$[Ag_6@(MoO_4)_7@Ag_{29}(^iPrS)_{16}Cl(H_2O)(C_3H_7NO)]^{2+}$	3111.6822	3111.6649
**G**	$[Ag_6@(MoO_4)_7@Ag_{30}(^iPrS)_{16}(p\text{-}TOS)Cl(H_2O)(C_3H_7NO)]^{2+}$	3251.6416	3251.6232

Supplementary Table 3: The assigned formula of species found in ESI-MS of **SD/Ag8** dissolved in CH_3CN.

**Peaks for SD/Ag8**	**Species**	**Exp. *m/z***	**Sim. *m/z***
**2a**	$[Ag_6@(MoO_4)_7@Ag_{32}(^iPrS)_5(p\text{-TOS})_{11}(OH)_2(CH_3CN)(H_2O)_4]^{4+}$	1906.2889	1906.2301
**2b**	$[Ag_6@(MoO_4)_7@Ag_{35}(^iPrS)_{11}(p\text{-TOS})_6Cl(OH)_3(CH_3CN)_4(H_2O)_9]^{4+}$	1952.2731	1952.2102
**2c**	$[Ag_6@(MoO_4)_7@Ag_{36}(^iPrS)_{16}(p\text{-TOS})_6(CH_3CN)(H_2O)_4]^{4+}$	1998.2529	1998.1917
**2d**	$[Ag_6@(MoO_4)_7@Ag_{36}(^iPrS)_7(p\text{-TOS})_{12}Cl_2(OH)(CH3CN)_2(H_2O)_6]^{4+}$	2127.1608	2127.1467
**2e**	$[Ag_6@(MoO_4)_7@Ag_{36}(^iPrS)_7(p\text{-TOS})_{12}Cl_3(CH_3CN)_3(H_2O)_5]^{4+}$	2137.6580	2137.6420

Supplementary Table 4: The assigned formula of species found in ESI-MS of **SD/Ag9** dissolved in CH_3CN.

**Peaks for SD/Ag9**	**Species**	**Exp.** ***m/z***	**Sim.** ***m/z***
**3a**	$[Ag_6@(MoO_4)_7@Ag_{32}(^iPrS)_8(CF_3SO_3)_{10}(H_2O)_2]^{4+}$	1836.8044	1836.8344
**3b**	$[Ag_6@(MoO_4)_7@Ag_{33}(^iPrS)_9(CF_3SO_3)_{10}(H_2O)_2]^{4+}$	1882.2848	1882.3174
**3c**	$[Ag_6@(MoO_4)_7@Ag_{34}(^iPrS)_{10}(CF_3SO_3)_{10}(H_2O)_2]^{4+}$	1928.2685	1928.3003
**3d**	$[Ag_6@(MoO_4)_7@Ag_{35}(^iPrS)_9(CF_3SO_3)_{10}Cl_2(CH_3CN)(H_2O)_2]^{4+}$	1964.2412	1964.2606
**3e**	$[Ag_6@(MoO_4)_7@Ag_{36}(^iPrS)_{13}(CF_3SO_3)_9(H_2O)_4]^{4+}$	2010.2228	2010.2902
**3f**	$[Ag_6@(MoO_4)_7@Ag_{37}(^iPrS)_{11}(CF_3SO_3)_{10}Cl_2(CH_3CN)(H_2O)_2]^{4+}$	2055.9542	2055.9764

Supplementary Table 5: The assigned formula of species found in ESI-MS of **SD/Ag11** dissolved in CH_3CN.

Peaks for SD/Ag11	Species	Exp. *m/z*	Sim. *m/z*
**5a**	$[Mo_7O_{24}@Ag_{41}(^iPrS)_{27}(p\text{-}TOS)_3Cl_2(CH_3CN)_2]^{3+}$	2724.6848	2724.6889
**5b**	$[Mo_7O_{24}@Ag_{41}(^iPrS)_{26}(p\text{-}TOS)_4Cl_2(CH_3CN)_2]^{3+}$	2756.6623	2756.6838
**5c**	$[Mo_7O_{24}@Ag_{41}(^iPrS)_{22}(p\text{-}TOS)_5Cl_3(OH)_2(CH_3CN)_3(H_2O)_6]^{3+}$	2786.3331	2786.3399
**5d**	$[Mo_7O_{24}@Ag_{41}(^iPrS)_{24}(p\text{-}TOS)_6Cl_2(H_2O)_4]^{3+}$	2817.3186	2817.3368
**5e**	$[Mo_7O_{24}@Ag_{41}(^iPrS)_{23}(p\text{-}TOS)_7(OH)_2(H_2O)_6]^{3+}$	2848.9803	2849.0286
**5f**	$[Mo_7O_{24}@Ag_{41}(^iPrS)_{19}(p\text{-}TOS)_8Cl_5(CH_3CN)(H_2O)_8]^{3+}$	2879.3025	2879.2911
**5g**	$[Mo_7O_{24}@Ag_{41}(^iPrS)_{18}(p\text{-}TOS)_9Cl_5(CH_3CN)_4(H_2O)]^{3+}$	2910.2894	2910.2880
**5h**	$[Mo_7O_{24}@Ag_{41}(^iPrS)_{18}(p\text{-}TOS)_{10}Cl_4(CH_3CN)_3(H_2O)]^{3+}$	2941.9487	2941.9603
**5i**	$[Mo_7O_{24}@Ag_{41}(^iPrS)_{17}(p\text{-}TOS)_{12}Cl_3]^{3+}$	2972.2652	2972.2730
**5j**	$[Mo_7O_{24}@Ag_{41}(^iPrS)_{17}(p\text{-}TOS)_{12}Cl_2(OH)(CH_3CN)(H_2O)_4]^{3+}$	3003.5908	3003.6409
**5k**	$[Mo_7O_{24}@Ag_{41}(^iPrS)_{14}(p\text{-}TOS)_{13}Cl_5(CH_3CN)_2(H_2O)_5]^{3+}$	3034.9211	3034.9309
**5l**	$[Mo_7O_{24}@Ag_{41}(^iPrS)_{15}(p\text{-}TOS)_{13}Cl_4(CH_3CN)_5(H_2O)]^{3+}$	3065.2257	3065.2963
**5m**	$[Mo_7O_{24}@Ag_{41}(^iPrS)_{12}(p\text{-}TOS)_{14}Cl_6(CH_3CN)_6(H_2O)_3]^{3+}$	3096.5581	3096.6014
**5n**	$[Mo_7O_{24}@Ag_{41}(^iPrS)_{11}(p\text{-}TOS)_{16}Cl_4(OH)(CH_3CN)_4(H_2O)]^{3+}$	3128.2121	3128.2644
**5o**	$[Mo_7O_{24}@Ag_{41}(^iPrS)_{11}(p\text{-}TOS)_{17}Cl_4(CH_3CN)_2(H_2O)_2]^{3+}$	3158.1924	3158.2531
**5p**	$[Mo_7O_{24}@Ag_{41}(^iPrS)_6(p\text{-}TOS)_{17}Cl_9(CH_3CN)_{10}]^{3+}$	3189.5269	3189.5527
**5q**	$[Mo_7O_{24}@Ag_{41}(p\text{-}TOS)_{21}Cl_8(OH)_3(CH_3CN)_4(H_2O)_5]^{3+}$	3220.8419	3220.8258
**5r**	$[Mo_7O_{24}@Ag_{41}(p\text{-}TOS)_{23}Cl_1(OH)_8(CH_3CN)(H_2O)_7]^{3+}$	3251.4839	3251.5596
**5s**	$[Mo_7O_{24}@Ag_{41}(p\text{-}TOS)_{23}Cl_7(OH)_2(CH_3CN)(H_2O)_6]^{3+}$	3282.1538	3282.1536
**5t**	$[Mo_7O_{24}@Ag_{41}(^iPrS)_{16}(p\text{-}TOS)_{11}Cl_5(OH)(CH_3CN)(H_2O)_6]^{2+}$	4453.4261	4453.4051
**5u**	$[Mo_7O_{24}@Ag_{41}(^iPrS)_{14}(p\text{-}TOS)_{12}Cl_5(OH)_2(CH_3CN)(H_2O)_9]^{2+}$	4499.4137	4499.4014
**5v**	$[Mo_7O_{24}@Ag_{41}(^iPrS)_{13}(p\text{-}TOS)_{13}Cl_3(OH)_4(CH_3CN)(H_2O)_{11}]^{2+}$	4546.9199	4546.9391
**5w**	$[Mo_7O_{24}@Ag_{41}(^iPrS)_{10}(p\text{-}TOS)_{15}Cl_6(OH)_2(CH_3CN)_3(H_2O)]^{2+}$	4592.8767	4592.8338
**5x**	$[Mo_7O_{24}@Ag_{41}(^iPrS)_{14}(p\text{-}TOS)_{15}Cl_4(CH_3CN)]^{2+}$	4640.3747	4640.3846
**5y**	$[Mo_7O_{24}@Ag_{41}(^iPrS)_{10}(p\text{-}TOS)_{15}Cl_7(OH)(CH_3CN)_5(H_2O)_6]^{2+}$	4687.8483	4687.8696
**5z**	$[Mo_7O_{24}@Ag_{41}(^iPrS)_8(p\text{-}TOS)_{18}Cl_6(OH)(CH_3CN)(H_2O)_2]^{2+}$	4733.8418	4733.8018

Supplementary Table 6: Selected bond distances (Å) and angles (°) for **SD/Ag7-SD/Ag12.**

SD/Ag7			
Ag1—Ag2	2.659 (4)	Ag15—S4	2.494 (4)
Ag1—Ag3	2.819 (4)	Ag15—S8	2.483 (4)
Ag1—Ag3^{i}	2.695 (4)	Ag16—Ag18	3.2601 (19)
Ag1—O26^{i}	2.342 (11)	Ag16—Ag23	2.9259 (19)
Ag1—O36	2.390 (10)	Ag16—O22	2.491 (13)
Ag2—Ag3^{i}	2.804 (4)	Ag16—S8	2.484 (4)
Ag2—Ag3	2.710 (4)	Ag16—S10	2.490 (4)
Ag2—O28	2.260 (10)	Ag17—Ag19	3.1164 (19)
Ag3—O29	2.560 (11)	Ag17—Ag26	3.120 (2)
Ag3—O33	2.367 (11)	Ag17—Ag28	2.971 (2)
Ag4—Ag10	3.022 (5)	Ag17—Ag31^{i}	3.136 (2)
Ag4—Ag11	2.954 (5)	Ag17—O24^{i}	2.460 (11)
Ag4—Ag18	3.189 (4)	Ag17—S2	2.471 (4)
Ag4—Ag21	3.132 (4)	Ag17—S16^{i}	2.527 (4)
Ag4—O12	2.451 (15)	Ag18—O30	2.545 (10)
Ag4—S7	2.517 (6)	Ag18—O32	2.562 (11)
Ag4—S11	2.517 (6)	Ag18—S7	2.391 (4)
Ag5—Ag8^{i}	3.192 (8)	Ag18—S10	2.393 (4)
Ag5—Ag13	3.063 (4)	Ag19—O6	2.475 (12)
Ag5—Ag19	3.262 (5)	Ag19—S2	2.502 (4)
Ag5—Ag29^{i}	3.030 (5)	Ag19—S19^{i}	2.499 (4)
Ag5—O5	2.555 (14)	Ag20—Ag21	3.108 (2)
Ag5—O35	2.593 (12)	Ag20—Ag27	3.191 (2)
Ag5—S3	2.407 (7)	Ag20—O28	2.391 (9)
Ag5—S19^{i}	2.543 (6)	Ag20—S11	2.503 (5)
Ag6—Ag16	2.978 (7)	Ag20—S12	2.492 (4)
Ag6—Ag23	3.203 (5)	Ag21—Ag22	3.182 (2)
Ag6—O29	2.312 (12)	Ag21—Ag27	2.981 (2)
Ag6—S8	2.488 (8)	Ag21—O30	2.541 (10)
Ag6—S14	2.373 (8)	Ag21—S11	2.482 (4)
Ag7—Ag25	3.326 (2)	Ag21—S13	2.471 (4)
Ag7—Ag27	3.251 (2)	Ag22—Ag23	3.143 (2)
Ag7—O15	2.511 (12)	Ag22—O27	2.543 (10)
Ag7—O18	2.583 (13)	Ag22—S10	2.459 (4)
Ag7—S13	2.506 (4)	Ag22—S13	2.460 (4)
Ag7—S17	2.506 (4)	Ag23—Ag25	3.1449 (19)
Ag8—Ag29	3.017 (5)	Ag23—O17	2.540 (14)

Ag8—O37^{i}	2.362 (12)	Ag23—O21	2.400 (12)
Ag8—S3^{i}	2.445 (9)	Ag23—S10	2.552 (4)
Ag8—S12	2.396 (8)	Ag23—S14	2.498 (4)
Ag9—Ag13	3.218 (3)	Ag24—Ag25	3.0816 (19)
Ag9—Ag28	3.183 (3)	Ag24—Ag31	3.072 (2)
Ag9—O7	2.519 (11)	Ag24—O26	2.359 (9)
Ag9—O8	2.569 (12)	Ag24—S14	2.501 (4)
Ag9—S2	2.518 (5)	Ag24—S16	2.496 (4)
Ag9—S4	2.509 (5)	Ag25—Ag31	2.961 (2)
Ag10—Ag11	3.1393 (19)	Ag25—O27	2.420 (11)
Ag10—Ag26	3.3664 (19)	Ag25—S14	2.493 (4)
Ag10—O1^{i}	2.58 (2)	Ag25—S17	2.441 (4)
Ag10—O31	2.315 (9)	Ag26—Ag28	3.149 (2)
Ag10—S6	2.449 (5)	Ag26—O1^{i}	2.45 (2)
Ag10—S11	2.445 (4)	Ag26—O36	2.368 (9)
Ag11—Ag12	3.108 (2)	Ag26—S6	2.472 (4)
Ag11—Ag28	3.3310 (19)	Ag26—S16^{i}	2.481 (5)
Ag11—O10	2.542 (17)	Ag27—Ag29	3.083 (2)
Ag11—O11	2.431 (13)	Ag27—O25	2.493 (11)
Ag11—S6	2.478 (4)	Ag27—S12	2.491 (5)
Ag11—S7	2.500 (4)	Ag27—S13	2.438 (4)
Ag12—Ag15	3.0878 (19)	Ag28—O34	2.400 (12)
Ag12—O34	2.544 (11)	Ag28—S2	2.456 (4)
Ag12—S4	2.471 (4)	Ag28—S6	2.503 (4)
Ag12—S7	2.461 (4)	Ag29—Ag30	3.124 (2)
Ag13—Ag14	3.154 (3)	Ag29—O25	2.439 (11)
Ag13—Ag15	2.993 (2)	Ag29—S12	2.499 (5)
Ag13—O35	2.483 (11)	Ag29—S19	2.448 (5)
Ag13—S3	2.509 (5)	Ag30—Ag31	3.065 (2)
Ag13—S4	2.404 (4)	Ag30—O14	2.469 (13)
Ag14—Ag15	3.118 (2)	Ag30—O40	2.500 (13)
Ag14—O33	2.344 (9)	Ag30—S17	2.493 (4)
Ag14—S3	2.502 (5)	Ag30—S19	2.502 (5)
Ag14—S8	2.495 (5)	Ag31—O24	2.480 (12)
Ag15—Ag16	3.2180 (19)	Ag31—S16	2.507 (4)
Ag15—O23	2.521 (14)	Ag31—S17	2.467 (4)
Ag15—O32	2.526 (10)		
O26^{i}—Ag1—O36	86.8 (4)	S10—Ag16—O22	98.2 (5)
O33—Ag3—O29	83.4 (3)	O24^{i}—Ag17—S2	112.9 (3)
O12—Ag4—S7	100.1 (4)	O24^{i}—Ag17—S16^{i}	85.5 (3)
O12—Ag4—S11	95.7 (5)	S2—Ag17—S16^{i}	158.57 (15)
S11—Ag4—S7	155.3 (2)	O30—Ag18—O32	71.7 (3)

O5—Ag5—O35	88.9 (4)	S7—Ag18—O30	104.2 (2)
S3—Ag5—O5	95.8 (4)	S7—Ag18—O32	88.5 (2)
S3—Ag5—O35	82.9 (3)	S7—Ag18—S10	165.84 (16)
S3—Ag5—S19^{i}	157.1 (3)	S10—Ag18—O30	87.6 (2)
S19^{i}—Ag5—O5	105.8 (4)	S10—Ag18—O32	102.9 (2)
S19^{i}—Ag5—O35	104.8 (3)	O6—Ag19—S2	98.3 (3)
O29—Ag6—S8	108.5 (4)	O6—Ag19—S19^{i}	100.1 (4)
O29—Ag6—S14	107.0 (4)	S19^{i}—Ag19—S2	148.91 (14)
S14—Ag6—S8	138.6 (4)	O28—Ag20—S11	100.8 (2)
O15—Ag7—O18	98.8 (4)	O28—Ag20—S12	103.4 (3)
S13—Ag7—O15	101.5 (3)	S12—Ag20—S11	138.51 (15)
S13—Ag7—O18	108.8 (4)	S11—Ag21—O30	84.9 (2)
S17—Ag7—O15	100.4 (3)	S13—Ag21—O30	106.2 (2)
S17—Ag7—O18	92.8 (4)	S13—Ag21—S11	163.44 (15)
S17—Ag7—S13	146.29 (15)	S10—Ag22—O27	104.6 (3)
O37^{i}—Ag8—S3^{i}	104.6 (4)	S10—Ag22—S13	167.26 (14)
O37^{i}—Ag8—S12	101.7 (4)	S13—Ag22—O27	83.5 (3)
S12—Ag8—S3^{i}	140.1 (3)	O17—Ag23—S10	110.8 (4)
O7—Ag9—O8	100.1 (4)	O21—Ag23—O17	89.3 (5)
S2—Ag9—O7	98.4 (3)	O21—Ag23—S10	95.8 (4)
S2—Ag9—O8	98.6 (3)	O21—Ag23—S14	106.4 (4)
S2—Ag9—S4	144.65 (16)	S14—Ag23—O17	95.7 (4)
S4—Ag9—O7	104.1 (3)	S14—Ag23—S10	145.57 (13)
S4—Ag9—O8	103.8 (3)	O26—Ag24—S14	105.2 (3)
O31—Ag10—O1^{i}	79.3 (5)	O26—Ag24—S16	101.2 (3)
O31—Ag10—S6	109.3 (3)	S16—Ag24—S14	139.22 (14)
O31—Ag10—S11	106.3 (3)	O27—Ag25—S14	89.6 (3)
S6—Ag10—O1^{i}	91.7 (5)	O27—Ag25—S17	107.0 (3)
S11—Ag10—O1^{i}	112.8 (5)	S17—Ag25—S14	159.90 (15)
S11—Ag10—S6	139.84 (14)	O1^{i}—Ag26—S6	94.2 (5)
O11—Ag11—O10	92.0 (4)	O1^{i}—Ag26—S16^{i}	122.5 (5)
O11—Ag11—S6	105.7 (4)	O36—Ag26—O1^{i}	83.7 (5)
O11—Ag11—S7	98.1 (4)	O36—Ag26—S6	107.8 (3)
S6—Ag11—O10	90.9 (4)	O36—Ag26—S16^{i}	100.8 (3)
S6—Ag11—S7	148.96 (14)	S6—Ag26—S16^{i}	135.59 (15)
S7—Ag11—O10	108.2 (4)	S12—Ag27—O25	84.9 (3)
S4—Ag12—O34	82.7 (3)	S13—Ag27—O25	107.0 (3)
S7—Ag12—O34	106.2 (3)	S13—Ag27—S12	159.91 (16)
S7—Ag12—S4	168.94 (15)	O34—Ag28—S2	112.9 (3)
O35—Ag13—S3	83.1 (3)	O34—Ag28—S6	87.1 (3)
S4—Ag13—O35	109.0 (3)	S2—Ag28—S6	155.91 (15)
S4—Ag13—S3	161.16 (17)	O25—Ag29—S12	85.9 (3)

O33—Ag14—S3	104.7 (3)	O25—Ag29—S19	112.8 (3)
O33—Ag14—S8	103.0 (3)	S19—Ag29—S12	159.87 (16)
S8—Ag14—S3	137.30 (16)	O14—Ag30—O40	89.2 (4)
O23—Ag15—O32	98.4 (5)	O14—Ag30—S17	101.7 (3)
S4—Ag15—O23	94.0 (5)	O14—Ag30—S19	96.0 (3)
S4—Ag15—O32	107.8 (3)	O40—Ag30—S19	92.1 (3)
S8—Ag15—O23	99.0 (4)	S17—Ag30—O40	104.5 (3)
S8—Ag15—O32	85.1 (2)	S17—Ag30—S19	155.73 (14)
S8—Ag15—S4	160.27 (15)	O24—Ag31—S16	85.5 (3)
S8—Ag16—O22	99.5 (5)	S17—Ag31—O24	112.9 (3)
S8—Ag16—S10	158.88 (14)	S17—Ag31—S16	158.85 (15)
Symmetry code: (i) $-x+2$, $-y+1$, $-z+2$.			
**SD/Ag8**			
Ag1A—Ag1B^{i}	2.772 (9)	Ag1N—S19	2.30 (3)
Ag1A—Ag1B	2.741 (7)	Ag1O—O29	2.56 (2)
Ag1A—Ag1C^{i}	2.682 (10)	Ag1O—O31	2.398 (18)
Ag1A—O1	2.49 (2)	Ag1O—S9	2.587 (13)
Ag1A—O16^{i}	2.219 (11)	Ag1O—S14	2.402 (13)
Ag1B—Ag1A^{i}	2.772 (9)	Ag1P—Ag1Q	3.362 (8)
Ag1B—Ag1C^{i}	2.702 (4)	Ag1P—O32	2.516 (15)
Ag1B—O2	2.47 (2)	Ag1P—O34	2.515 (18)
Ag1B—O9	2.349 (12)	Ag1P—S9	2.555 (7)
Ag1B—O13	2.475 (10)	Ag1P—S12	2.427 (7)
Ag1C—Ag1A^{i}	2.682 (10)	Ag1Q—Ag1D^{i}	2.895 (8)
Ag1C—Ag1B^{i}	2.702 (4)	Ag1Q—Ag1R	3.258 (10)
Ag1C—O3^{i}	2.44 (2)	Ag1Q—O6	2.495 (14)
Ag1C—O7	2.332 (12)	Ag1Q—O36	2.510 (16)
Ag1C—O15^{i}	2.495 (12)	Ag1Q—S1	2.470 (8)
Ag1D—Ag1E	3.316 (7)	Ag1Q—S9	2.561 (7)
Ag1D—Ag1Q^{i}	2.895 (8)	Ag1R—Ag1S	2.892 (11)
Ag1D—Ag24	3.307 (6)	Ag1R—O30	2.499 (16)
Ag1D—Ag25^{i}	3.063 (7)	Ag1R—S2	2.355 (10)
Ag1D—O6^{i}	2.557 (13)	Ag1R—S9	2.630 (9)
Ag1D—O36^{i}	2.507 (16)	Ag1S—Ag1T	3.325 (8)
Ag1D—S1^{i}	2.481 (8)	Ag1S—Ag1X	3.331 (6)
Ag1D—S11^{i}	2.523 (8)	Ag1S—Ag29	3.117 (6)
Ag1E—Ag1F	2.891 (13)	Ag1S—O11	2.565 (14)
Ag1E—Ag24	3.337 (4)	Ag1S—O30	2.497 (17)
Ag1E—Ag26	3.245 (4)	Ag1S—S2	2.492 (8)
Ag1E—O17	2.571 (12)	Ag1S—S7	2.506 (7)
Ag1E—O21	2.421 (13)	Ag1T—Ag1U	2.943 (11)
Ag1E—S11^{i}	2.496 (6)	Ag1T—Ag1X	3.288 (6)

Ag1E—S18	2.524 (6)	Ag1T—Ag28	3.116 (7)
Ag1F—Ag1G	3.100 (14)	Ag1T—O14	2.574 (13)
Ag1F—Ag27	3.144 (13)	Ag1T—O26	2.527 (16)
Ag1F—O17	2.534 (15)	Ag1T—S3	2.510 (8)
Ag1F—O21	2.590 (15)	Ag1T—S7	2.506 (8)
Ag1F—S16	2.489 (13)	Ag1U—Ag1V	3.178 (10)
Ag1F—S18	2.486 (14)	Ag1U—Ag27	3.254 (9)
Ag1G—Ag1H	3.084 (9)	Ag1U—Ag30	3.250 (8)
Ag1G—Ag1N	3.34 (4)	Ag1U—O26	2.458 (14)
Ag1G—Ag28	3.235 (7)	Ag1U—S3	2.455 (10)
Ag1G—O12	2.576 (13)	Ag1U—S6	2.534 (10)
Ag1G—O23	2.471 (13)	Ag1V—Ag1K^{i}	3.020 (16)
Ag1G—S16	2.456 (9)	Ag1V—Ag26	3.152 (7)
Ag1G—S19	2.529 (8)	Ag1V—Ag30	3.356 (6)
Ag1H—Ag1I	2.980 (8)	Ag1V—O37^{i}	2.500 (14)
Ag1H—Ag1M	3.029 (6)	Ag1V—S4	2.532 (8)
Ag1H—Ag29	3.282 (5)	Ag1V—S6	2.432 (7)
Ag1H—O12	2.537 (12)	Ag1W—O2	2.46 (3)
Ag1H—O23	2.572 (12)	Ag1W—O9	2.462 (18)
Ag1H—S14	2.455 (7)	Ag1W—S1	2.453 (15)
Ag1H—S19	2.527 (7)	Ag1W—S2	2.498 (15)
Ag1I—Ag1J	3.153 (11)	Ag1X—O13	2.338 (10)
Ag1I—Ag1M	3.071 (8)	Ag1X—S2	2.462 (5)
Ag1I—Ag1O	2.935 (12)	Ag1X—S3	2.459 (5)
Ag1I—O8	2.585 (13)	Ag24—O18	2.382 (10)
Ag1I—O33	2.503 (15)	Ag24—S1^{i}	2.466 (6)
Ag1I—S14	2.436 (9)	Ag24—S18	2.449 (5)
Ag1I—S20	2.535 (9)	Ag25—Ag1D^{i}	3.063 (7)
Ag1J—Ag1K	2.993 (16)	Ag25—O35	2.520 (15)
Ag1J—Ag1L	3.089 (6)	Ag25—O39	2.455 (13)
Ag1J—Ag1P	3.178 (9)	Ag25—S11	2.507 (5)
Ag1J—O8	2.526 (16)	Ag25—S12	2.488 (5)
Ag1J—O33	2.572 (14)	Ag26—O19	2.473 (12)
Ag1J—S12	2.491 (9)	Ag26—S6	2.487 (5)
Ag1J—S20	2.503 (9)	Ag26—S11^{i}	2.471 (5)
Ag1K—Ag1L	3.080 (15)	Ag27—O20	2.524 (12)
Ag1K—Ag1V^{i}	3.020 (16)	Ag27—O25	2.476 (13)
Ag1K—Ag25	3.240 (14)	Ag27—S6	2.472 (4)
Ag1K—O5	2.570 (18)	Ag27—S16	2.481 (4)
Ag1K—O37	2.528 (19)	Ag28—O22	2.484 (11)
Ag1K—S4^{i}	2.466 (15)	Ag28—O27	2.555 (12)
Ag1K—S12	2.497 (15)	Ag28—S7	2.494 (4)

Ag1L—O7	2.368 (11)	Ag28—S16	2.475 (4)
Ag1L—S4^{i}	2.418 (6)	Ag29—O24	2.499 (11)
Ag1L—S20	2.451 (5)	Ag29—O28	2.489 (12)
Ag1M—O10	2.314 (12)	Ag29—S7	2.499 (5)
Ag1M—S19	2.430 (8)	Ag29—S14	2.488 (5)
Ag1M—S20	2.437 (8)	Ag30—O3	2.510 (16)
Ag1N—O1^{i}	2.57 (5)	Ag30—O15	2.374 (10)
Ag1N—O16	2.49 (3)	Ag30—S3	2.462 (5)
Ag1N—S18	2.62 (3)	Ag30—S4	2.476 (5)
O16^{i}—Ag1A—O1	93.4 (7)	S12—Ag1P—O32	99.4 (4)
O2—Ag1B—O13	83.6 (6)	S12—Ag1P—O34	100.9 (4)
O9—Ag1B—O2	83.6 (7)	S12—Ag1P—S9	145.9 (3)
O9—Ag1B—O13	85.4 (4)	O6—Ag1Q—O36	92.7 (5)
O3^{i}—Ag1C—O15^{i}	81.4 (4)	O6—Ag1Q—S9	105.0 (3)
O7—Ag1C—O3^{i}	86.9 (5)	O36—Ag1Q—S9	106.8 (4)
O7—Ag1C—O15^{i}	83.7 (4)	S1—Ag1Q—O6	84.4 (3)
O36^{i}—Ag1D—O6^{i}	91.3 (5)	S1—Ag1Q—O36	93.7 (4)
O36^{i}—Ag1D—S11^{i}	109.6 (4)	S1—Ag1Q—S9	156.7 (3)
S1^{i}—Ag1D—O6^{i}	82.9 (3)	O30—Ag1R—S9	105.1 (5)
S1^{i}—Ag1D—O36^{i}	93.5 (4)	S2—Ag1R—O30	97.3 (5)
S1^{i}—Ag1D—S11^{i}	154.1 (3)	S2—Ag1R—S9	157.3 (4)
S11^{i}—Ag1D—O6^{i}	107.2 (3)	O30—Ag1S—O11	93.8 (5)
O21—Ag1E—O17	93.1 (4)	S2—Ag1S—O11	82.0 (3)
O21—Ag1E—S11^{i}	107.8 (3)	S2—Ag1S—O30	93.9 (4)
O21—Ag1E—S18	97.0 (3)	S2—Ag1S—S7	152.8 (3)
S11^{i}—Ag1E—O17	103.7 (3)	S7—Ag1S—O11	106.4 (3)
S11^{i}—Ag1E—S18	153.7 (2)	O26—Ag1T—O14	90.3 (4)
S18—Ag1E—O17	82.9 (3)	S3—Ag1T—O14	82.4 (3)
O17—Ag1F—O21	90.0 (5)	S3—Ag1T—O26	92.7 (4)
S16—Ag1F—O17	107.5 (5)	S7—Ag1T—O14	106.2 (3)
S16—Ag1F—O21	105.1 (5)	S7—Ag1T—O26	110.8 (4)
S18—Ag1F—O17	84.4 (4)	S7—Ag1T—S3	154.6 (3)
S18—Ag1F—O21	93.8 (5)	O26—Ag1U—S6	105.1 (5)
S18—Ag1F—S16	157.4 (6)	S3—Ag1U—O26	95.7 (5)
O23—Ag1G—O12	88.6 (5)	S3—Ag1U—S6	158.3 (4)
O23—Ag1G—S19	91.8 (4)	O37^{i}—Ag1V—S4	92.3 (4)
S16—Ag1G—O12	106.6 (4)	S6—Ag1V—O37^{i}	111.0 (4)
S16—Ag1G—O23	108.4 (4)	S6—Ag1V—S4	155.8 (3)
S16—Ag1G—S19	158.3 (4)	O2—Ag1W—O9	81.6 (7)
S19—Ag1G—O12	81.1 (3)	O2—Ag1W—S2	93.0 (8)
O12—Ag1H—O23	87.3 (4)	O9—Ag1W—S2	95.7 (5)
S14—Ag1H—O12	107.5 (3)	S1—Ag1W—O2	129.4 (8)

S14—Ag1H—O23	106.2 (3)	S1—Ag1W—O9	100.3 (6)
S14—Ag1H—S19	161.7 (2)	S1—Ag1W—S2	136.2 (6)
S19—Ag1H—O12	81.9 (3)	O13—Ag1X—S2	101.9 (3)
S19—Ag1H—O23	89.6 (3)	O13—Ag1X—S3	101.3 (3)
O33—Ag1I—O8	86.4 (4)	S3—Ag1X—S2	141.17 (18)
O33—Ag1I—S20	88.8 (4)	O18^{i}—Ag3A—O9	83.6 (4)
S14—Ag1I—O8	108.5 (4)	O18—Ag24—S1^{i}	100.5 (3)
S14—Ag1I—O33	108.5 (4)	O18—Ag24—S18	100.8 (3)
S14—Ag1I—S20	160.6 (4)	S18—Ag24—S1^{i}	141.56 (17)
S20—Ag1I—O8	80.6 (4)	O39—Ag25—O35	93.0 (5)
O8—Ag1J—O33	86.2 (5)	O39—Ag25—S11	98.9 (4)
S12—Ag1J—O8	108.2 (4)	O39—Ag25—S12	95.6 (4)
S12—Ag1J—O33	107.4 (4)	S11—Ag25—O35	97.2 (4)
S12—Ag1J—S20	161.5 (3)	S12—Ag25—O35	102.7 (4)
S20—Ag1J—O8	82.3 (4)	S12—Ag25—S11	154.61 (15)
S20—Ag1J—O33	88.0 (4)	O19—Ag26—S6	99.1 (3)
O37—Ag1K—O5	89.2 (6)	S11^{i}—Ag26—O19	98.5 (4)
S4^{i}—Ag1K—O5	84.5 (5)	S11^{i}—Ag26—S6	149.21 (16)
S4^{i}—Ag1K—O37	93.2 (6)	O25—Ag27—O20	94.6 (4)
S4^{i}—Ag1K—S12	161.0 (7)	O25—Ag27—S16	99.2 (3)
S12—Ag1K—O5	106.6 (6)	S6—Ag27—O20	104.7 (3)
S12—Ag1K—O37	102.2 (6)	S6—Ag27—O25	96.8 (3)
O7—Ag1L—S4^{i}	103.2 (3)	S6—Ag27—S16	153.83 (15)
O7—Ag1L—S20	103.8 (3)	S16—Ag27—O20	94.5 (3)
S4^{i}—Ag1L—S20	141.40 (19)	O22—Ag28—O27	100.3 (4)
O10—Ag1M—S19	104.1 (4)	O22—Ag28—S7	96.7 (3)
O10—Ag1M—S20	104.9 (4)	S7—Ag28—O27	94.9 (3)
S19—Ag1M—S20	143.5 (3)	S16—Ag28—O22	98.0 (3)
O1^{i}—Ag1N—S18	119.6 (15)	S16—Ag28—O27	106.1 (3)
O16—Ag1N—O1^{i}	85.3 (12)	S16—Ag28—S7	151.72 (15)
O16—Ag1N—S18	94.2 (7)	O28—Ag29—O24	101.3 (4)
S19—Ag1N—O1^{i}	102.6 (12)	O28—Ag29—S7	100.6 (3)
S19—Ag1N—O16	102.3 (14)	S7—Ag29—O24	105.7 (3)
S19—Ag1N—S18	135.8 (17)	S14—Ag29—O24	92.6 (3)
O29—Ag1O—S9	90.7 (5)	S14—Ag29—O28	100.9 (3)
O31—Ag1O—O29	93.8 (7)	S14—Ag29—S7	148.27 (15)
O31—Ag1O—S9	100.7 (6)	O15—Ag30—O3	82.5 (6)
O31—Ag1O—S14	104.5 (6)	O15—Ag30—S3	101.0 (3)
S14—Ag1O—O29	98.2 (5)	O15—Ag30—S4	101.9 (3)
S14—Ag1O—S9	152.5 (6)	S3—Ag30—O3	123.7 (4)
O32—Ag1P—S9	104.4 (4)	S3—Ag30—S4	138.81 (18)
O34—Ag1P—O32	105.1 (5)	S4—Ag30—O3	93.0 (4)

O34—Ag1P—S9	96.0 (4)		
Symmetry code: (i) −*x*+3/2, −*y*+3/2, −*z*+2.			
**SD/Ag9**			
Ag1—Ag2^{i}	2.522 (9)	Ag9—O4	2.299 (12)
Ag1—Ag3^{ii}	2.267 (4)	Ag9—S1	2.607 (6)
Ag1—O1	2.362 (11)	Ag9—S3	2.421 (6)
Ag1—O7	2.331 (14)	Ag10—Ag11	3.065 (4)
Ag1—O10^{i}	2.512 (19)	Ag10—O16	2.593 (13)
Ag2—Ag1^{i}	2.522 (9)	Ag10—S3	2.502 (5)
Ag2—Ag2^{ii}	2.42 (2)	Ag10—S10	2.470 (5)
Ag2—O2	2.405 (13)	Ag11—Ag12	2.986 (4)
Ag2—O11	2.42 (2)	Ag11—Ag17	3.058 (4)
Ag3—Ag1^{ii}	2.267 (4)	Ag11—O5	2.493 (11)
Ag3—Ag3^{iii}	2.179 (7)	Ag11—S6	2.465 (6)
Ag3—O2^{ii}	2.553 (10)	Ag11—S10	2.436 (6)
Ag3—O6^{ii}	2.345 (12)	Ag12—Ag14	3.109 (6)
Ag4—Ag5	3.150 (6)	Ag12—Ag17	3.092 (3)
Ag4—Ag17	3.337 (3)	Ag12—O23	2.51 (2)
Ag4—O7	2.292 (15)	Ag12—S10	2.554 (5)
Ag4—S6^{iii}	2.430 (6)	Ag12—S11	2.472 (5)
Ag4—S6	2.430 (6)	Ag13—Ag15	3.193 (7)
Ag5—Ag5^{iii}	2.854 (6)	Ag13—O13	2.523 (14)
Ag5—Ag6	3.119 (4)	Ag13—S9	2.461 (4)
Ag5—Ag11	3.146 (5)	Ag13—S10	2.487 (5)
Ag5—O5	2.413 (11)	Ag14—Ag15^{ii}	3.049 (9)
Ag5—S4	2.408 (7)	Ag14—Ag16^{ii}	3.250 (5)
Ag5—S6	2.556 (6)	Ag14—Ag18	3.247 (5)
Ag6—Ag7	3.056 (3)	Ag14—S9^{ii}	2.380 (7)
Ag6—S3	2.482 (5)	Ag14—S11	2.559 (7)
Ag6—S4	2.486 (5)	Ag15—Ag16	3.084 (6)
Ag7—Ag7^{iii}	3.140 (4)	Ag15—O4	2.575 (12)
Ag7—Ag8	3.075 (4)	Ag15—S1	2.444 (9)
Ag7—Ag9	2.815 (5)	Ag15—S9	2.481 (9)
Ag7—O8	2.490 (12)	Ag16—O2	2.399 (9)
Ag7—S2	2.509 (5)	Ag16—O11	2.42 (3)
Ag7—S3	2.436 (5)	Ag16—S1	2.482 (5)
Ag8—Ag8^{iii}	3.295 (5)	Ag16—S11^{ii}	2.495 (4)
Ag8—Ag9	2.909 (5)	Ag17—O1	2.356 (9)
Ag8—O6	2.317 (11)	Ag17—O10^{i}	2.49 (2)
Ag8—S1	2.413 (5)	Ag17—S6	2.473 (5)
Ag8—S2	2.431 (6)	Ag17—S11	2.468 (5)
Ag9—Ag10	3.151 (4)	Ag18—O12	2.583 (14)

Ag9—Ag15	3.163 (9)	Ag18—S9	2.484 (4)
O1—Ag1—O10^{i}	82.7 (5)	O23—Ag12—S10	102.2 (8)
O7—Ag1—O1	85.7 (3)	S11—Ag12—O23	88.1 (8)
O7—Ag1—O10^{i}	84.9 (6)	S11—Ag12—S10	159.22 (18)
O2—Ag2—O11	84.9 (7)	S9—Ag13—O13	104.3 (3)
O6^{ii}—Ag3—O2^{ii}	83.0 (4)	S9—Ag13—S10	159.39 (17)
O7—Ag4—S6^{iii}	106.7 (2)	S10—Ag13—O13	88.7 (3)
O7—Ag4—S6	106.7 (2)	S9^{ii}—Ag14—S11	158.0 (3)
S6^{iii}—Ag4—S6	132.0 (4)	S1—Ag15—O4	85.4 (3)
O5—Ag5—S6	83.5 (3)	S1—Ag15—S9	162.5 (3)
S4—Ag5—O5	116.6 (3)	S9—Ag15—O4	100.6 (3)
S4—Ag5—S6	156.8 (2)	O2—Ag16—O11	85.2 (6)
O8—Ag7—S2	82.1 (3)	O2—Ag16—S1	104.0 (3)
S3—Ag7—O8	112.6 (3)	O2—Ag16—S11^{ii}	97.9 (3)
S3—Ag7—S2	158.59 (17)	O11—Ag16—S1	121.2 (9)
O6—Ag8—S1	100.2 (3)	O11—Ag16—S11^{ii}	90.4 (9)
O6—Ag8—S2	111.3 (3)	S1—Ag16—S11^{ii}	142.44 (16)
S1—Ag8—S2	138.7 (2)	O1—Ag17—O10^{i}	83.3 (5)
O4—Ag9—S1	87.7 (3)	O1—Ag17—S6	107.6 (3)
O4—Ag9—S3	119.3 (3)	O1—Ag17—S11	100.2 (3)
S3—Ag9—S1	152.8 (2)	S6—Ag17—O10^{i}	96.9 (5)
S3—Ag10—O16	91.4 (3)	S11—Ag17—O10^{i}	113.4 (5)
S10—Ag10—O16	104.0 (3)	S11—Ag17—S6	140.89 (15)
S10—Ag10—S3	154.35 (17)	O12^{ii}—Ag18—O12	89.9 (6)
S6—Ag11—O5	83.8 (3)	S9—Ag18—O12^{ii}	91.5 (4)
S10—Ag11—O5	109.0 (3)	S9—Ag18—O12	102.2 (4)
S10—Ag11—S6	161.6 (2)	S9^{ii}—Ag18—S9	160.6 (2)
Symmetry codes: (i) −*x*+1, −*y*+1, −*z*+2; (ii) −*y*+1, −*x*+1, −*z*+2; (iii) *y*, *x*, *z*.			
**SD/Ag10**			
Ag1—Ag7	2.837 (9)	Ag9—O1	2.431 (16)
Ag1—Ag13	3.298 (7)	Ag9—O10	2.356 (8)
Ag1—Ag14	3.287 (6)	Ag9—O57	2.38 (4)
Ag1—Ag16	3.098 (3)	Ag9—S2	2.463 (5)
Ag1—O14	2.499 (18)	Ag9—S3	2.506 (5)
Ag1—S6	2.482 (5)	Ag10—Ag6^{iii}	2.603 (5)
Ag1—S7	2.494 (5)	Ag10—Ag6^{i}	2.438 (4)
Ag2—Ag2^{i}	3.122 (7)	Ag10—Ag6^{ii}	3.019 (4)
Ag2—Ag3	3.074 (10)	Ag10—O2^{iii}	2.422 (17)
Ag2—Ag14	3.127 (4)	Ag10—O4	2.290 (12)
Ag2—O5	2.328 (10)	Ag10—O8^{i}	2.518 (10)
Ag2—S5	2.370 (5)	Ag11—Ag18	2.874 (3)
Ag2—S8	2.479 (5)	Ag11—O11^{ii}	2.473 (10)

Ag3—Ag4	2.957 (7)	Ag11—S1	2.458 (5)
Ag3—Ag12	3.183 (4)	Ag11—S2	2.467 (5)
Ag3—S4	2.517 (8)	Ag12—Ag12^{i}	3.369 (4)
Ag3—S5	2.481 (6)	Ag12—O1	2.597 (17)
Ag4—Ag8	3.237 (12)	Ag12—O7	2.320 (8)
Ag4—Ag12	3.299 (5)	Ag12—S3	2.458 (6)
Ag4—Ag13	3.202 (8)	Ag12—S4	2.435 (7)
Ag4—S3	2.450 (6)	Ag13—S5	2.429 (5)
Ag4—S5	2.526 (6)	Ag13—S6	2.427 (5)
Ag5—Ag8	3.227 (9)	Ag14—Ag14^{i}	2.903 (3)
Ag5—S1	2.418 (6)	Ag14—Ag15	3.091 (2)
Ag5—S6	2.508 (7)	Ag14—O6	2.558 (9)
Ag6—O1	2.466 (14)	Ag14—S7	2.465 (5)
Ag6—O7	2.384 (10)	Ag14—S8	2.469 (6)
Ag6—O10	2.415 (9)	Ag15—O2^{iii}	2.432 (19)
Ag7—Ag16	3.086 (5)	Ag15—O2^{ii}	2.432 (19)
Ag7—O21	2.51 (3)	Ag15—O4	2.363 (12)
Ag7—S2^{ii}	2.536 (7)	Ag15—S7	2.484 (4)
Ag7—S6	2.482 (7)	Ag16—O8	2.268 (9)
Ag8—Ag9	2.939 (8)	Ag16—S2^{ii}	2.450 (6)
Ag8—Ag11	2.735 (9)	Ag16—S7	2.439 (5)
Ag8—O9	2.323 (12)	Ag17—Ag18	1.025 (6)
Ag8—S1	2.406 (8)	Ag17—S1	2.558 (6)
Ag8—S3	2.553 (9)	Ag18—S1	2.386 (5)
Ag9—Ag11	3.140 (3)		
S6—Ag1—O14	106.5 (5)	S1—Ag11—S2	161.8 (2)
S6—Ag1—S7	149.7 (3)	S2—Ag11—O11^{ii}	85.6 (2)
S7—Ag1—O14	103.0 (5)	O7—Ag12—O1	84.2 (4)
O5—Ag2—S5	112.2 (3)	O7—Ag12—S3	103.1 (2)
O5—Ag2—S8	87.0 (3)	O7—Ag12—S4	108.9 (3)
S5—Ag2—S8	160.5 (3)	S3—Ag12—O1	87.7 (4)
S5—Ag3—S4	151.1 (4)	S4—Ag12—O1	125.9 (4)
S3—Ag4—S5	150.0 (3)	S4—Ag12—S3	134.90 (19)
S1—Ag5—S6	159.4 (6)	S6—Ag13—S5	176.48 (16)
O7—Ag6—O1	85.8 (4)	S7—Ag14—O6	85.1 (3)
O7—Ag6—O10	84.3 (3)	S7—Ag14—S8	163.80 (15)
O10—Ag6—O1	83.8 (4)	S8—Ag14—O6	101.3 (3)
O21—Ag7—S2^{ii}	103.4 (7)	O2^{iii}—Ag15—O2^{ii}	27.7 (11)
S6—Ag7—O21	104.1 (7)	O2^{ii}—Ag15—S7	94.4 (5)
S6—Ag7—S2^{ii}	148.3 (2)	O2^{iii}—Ag15—S7^{i}	94.4 (5)
O9—Ag8—S1	111.0 (4)	O2^{iii}—Ag15—S7	121.2 (6)
O9—Ag8—S3	91.2 (3)	O2^{ii}—Ag15—S7^{i}	121.2 (6)

S1—Ag8—S3	156.7 (3)	O4—Ag15—O2^{iii}	84.2 (5)
O1—Ag9—S2	128.3 (4)	O4—Ag15—O2^{ii}	84.2 (5)
O1—Ag9—S3	90.4 (4)	O4—Ag15—S7^{i}	105.86 (13)
O10—Ag9—O1	85.8 (4)	O4—Ag15—S7	105.86 (13)
O10—Ag9—O57	87.1 (9)	S7^{i}—Ag15—S7	134.1 (2)
O10—Ag9—S2	105.4 (3)	O8—Ag16—S2^{ii}	108.6 (3)
O10—Ag9—S3	100.9 (3)	O8—Ag16—S7	108.9 (3)
O57—Ag9—O1	20.8 (9)	S7—Ag16—S2^{ii}	138.72 (15)
O57—Ag9—S2	108.0 (10)	S1^{ii}—Ag17—S1	137.5 (3)
O57—Ag9—S3	110.5 (10)	S1^{ii}—Ag18—S1	175.3 (3)
S2—Ag9—S3	133.92 (17)	O4—Ag10—O8^{i}	83.4 (2)
O2^{iii}—Ag10—O8^{i}	83.3 (6)	S1—Ag11—O11^{ii}	105.3 (3)
O4—Ag10—O2^{iii}	86.0 (6)		
Symmetry codes: (i) x, $-y$+1, z; (ii) $-x$+1, y, $-z$+1; (iii) $-x$+1, $-y$+1, $-z$+1.			
**SD/Ag11**			
Ag1—Ag4	3.0842 (10)	Ag10—S3	2.480 (3)
Ag1—Ag14^{i}	3.0679 (11)	Ag10—S5	2.474 (3)
Ag1—O10	2.446 (6)	Ag11—Ag12	3.2830 (11)
Ag1—O26	2.577 (8)	Ag11—Ag16	2.9773 (10)
Ag1—S1^{i}	2.532 (2)	Ag11—O37	2.488 (7)
Ag1—S7	2.527 (2)	Ag11—S2	2.480 (2)
Ag2—Ag3	3.2860 (11)	Ag11—S5	2.474 (2)
Ag2—Ag5	3.3077 (11)	Ag12—O32	2.425 (6)
Ag2—Ag6	3.0741 (12)	Ag12—O36	2.578 (8)
Ag2—O25	2.498 (7)	Ag12—S2	2.491 (2)
Ag2—O27	2.463 (8)	Ag12—S6	2.510 (2)
Ag2—S7	2.456 (2)	Ag13—Ag14	3.1051 (9)
Ag2—S8	2.461 (3)	Ag13—S6	2.388 (2)
Ag3—O28	2.337 (9)	Ag14—Ag1^{i}	3.0679 (11)
Ag3—O30	2.467 (6)	Ag14—Ag4^{i}	2.9788 (11)
Ag3—S8	2.485 (3)	Ag14—Ag15	3.2019 (10)
Ag4—Ag5	3.3739 (11)	Ag14—O34	2.591 (7)
Ag4—Ag12	3.2087 (10)	Ag14—S1	2.452 (2)
Ag4—Ag14^{i}	2.9789 (11)	Ag14—S6^{i}	2.445 (2)
Ag4—O9	2.405 (6)	Ag15—Ag21	3.1507 (11)
Ag4—O36	2.585 (7)	Ag15—S1	2.482 (2)
Ag4—S6	2.483 (2)	Ag15—S2	2.474 (2)
Ag4—S7	2.433 (2)	Ag16—O14	2.376 (8)
Ag5—Ag6	3.0734 (11)	Ag16—O40	2.567 (6)
Ag5—O24	2.495 (7)	Ag16—S2	2.503 (2)
Ag5—O35	2.461 (7)	Ag16—S3	2.499 (3)
Ag5—S5	2.521 (3)	Ag17—Ag18	3.0945 (11)

Ag5—S7	2.519 (3)	Ag17—O15	2.405 (8)
Ag6—O22	2.469 (8)	Ag17—O16	2.456 (8)
Ag6—O23	2.537 (8)	Ag17—S3	2.522 (3)
Ag6—S5	2.518 (3)	Ag17—S4	2.526 (3)
Ag6—S8	2.495 (3)	Ag18—Ag19	2.8855 (11)
Ag7—S8	2.443 (3)	Ag18—O19	2.396 (7)
Ag7—S9	2.442 (3)	Ag18—S4	2.484 (3)
Ag8—Ag18	3.0406 (12)	Ag18—S9	2.502 (3)
Ag8—Ag19	3.1818 (11)	Ag19—Ag20	3.0681 (11)
Ag8—O8	2.569 (6)	Ag19—O2	2.519 (6)
Ag8—O31	2.551 (7)	Ag19—O31^{i}	2.551 (7)
Ag8—S9	2.461 (3)	Ag19—S4	2.469 (3)
Ag8—S18	2.4782 (12)	Ag19—S18	2.4729 (12)
Ag9—Ag10	3.1076 (11)	Ag20—O7^{i}	2.496 (6)
Ag9—Ag18	2.9362 (11)	Ag20—O12	2.351 (7)
Ag9—O18	2.431 (7)	Ag20—O30^{i}	2.411 (7)
Ag9—O21	2.475 (9)	Ag20—S4	2.462 (3)
Ag9—S3	2.457 (3)	Ag21—O1	2.377 (6)
Ag9—S9	2.516 (3)	Ag21—O12	2.452 (7)
Ag10—Ag11	3.0764 (10)	Ag21—O13	2.370 (8)
Ag10—Ag16	2.9295 (10)	Ag21—S1	2.498 (2)
Ag10—O11	2.588 (6)		
O10—Ag1—O26	128.2 (2)	S5—Ag10—S3	155.94 (9)
O10—Ag1—S1^{i}	101.81 (15)	S2—Ag11—O37	100.9 (2)
O10—Ag1—S7	100.21 (15)	S5—Ag11—O37	98.2 (2)
S1^{i}—Ag1—O26	89.84 (19)	S5—Ag11—S2	160.98 (8)
S7—Ag1—O26	95.8 (2)	O32—Ag12—O36	110.4 (2)
S7—Ag1—S1^{i}	146.79 (8)	O32—Ag12—S2	93.89 (18)
O27—Ag2—O25	88.4 (2)	O32—Ag12—S6	101.30 (18)
S7—Ag2—O25	105.14 (17)	S2—Ag12—O36	116.90 (18)
S7—Ag2—O27	108.8 (2)	S2—Ag12—S6	151.41 (8)
S7—Ag2—S8	138.83 (9)	S6—Ag12—O36	80.29 (18)
S8—Ag2—O25	103.13 (18)	S6—Ag13—S6^{i}	175.53 (12)
S8—Ag2—O27	101.2 (2)	S1—Ag14—O34	83.27 (17)
O28—Ag3—O30	89.6 (2)	S6^{i}—Ag14—O34	103.91 (16)
O28—Ag3—S8	127.71 (19)	S6^{i}—Ag14—S1	165.09 (8)
O30—Ag3—S8	128.05 (18)	S2—Ag15—S1	152.11 (8)
O9—Ag4—O36	89.7 (2)	O14—Ag16—O40	126.2 (3)
O9—Ag4—S6	102.12 (16)	O14—Ag16—S2	96.5 (2)
O9—Ag4—S7	94.51 (16)	O14—Ag16—S3	92.6 (2)
S6—Ag4—O36	80.68 (18)	S2—Ag16—O40	77.02 (14)
S7—Ag4—O36	113.54 (18)	S3—Ag16—O40	104.82 (15)

S7—Ag4—S6	158.31 (9)	S3—Ag16—S2	167.07 (9)
O24—Ag5—S5	111.0 (2)	O15—Ag17—O16	81.0 (3)
O24—Ag5—S7	98.41 (19)	O15—Ag17—S3	98.5 (2)
O35—Ag5—O24	95.2 (2)	O15—Ag17—S4	114.0 (2)
O35—Ag5—S5	97.7 (2)	O16—Ag17—S3	115.4 (3)
O35—Ag5—S7	111.47 (19)	O16—Ag17—S4	90.3 (3)
S7—Ag5—S5	136.28 (8)	S4—Ag17—S3	141.67 (9)
O22—Ag6—O23	74.4 (3)	O19—Ag18—S4	101.2 (2)
O22—Ag6—S5	90.4 (2)	O19—Ag18—S9	96.8 (2)
O22—Ag6—S8	115.6 (2)	S4—Ag18—S9	161.39 (8)
S5—Ag6—O23	103.3 (2)	O2—Ag19—O31^{i}	100.2 (2)
S8—Ag6—O23	104.1 (2)	S4—Ag19—O2	98.02 (15)
S8—Ag6—S5	146.30 (9)	S4—Ag19—O31^{i}	113.18 (17)
S8—Ag7—S9	162.40 (9)	S4—Ag19—S18	158.92 (7)
O31—Ag8—O8	72.3 (2)	S18—Ag19—O2	93.17 (15)
S9—Ag8—O8	97.49 (15)	S18—Ag19—O31^{i}	82.04 (16)
S9—Ag8—O31	116.52 (16)	O12—Ag20—O7^{i}	74.1 (2)
S9—Ag8—S18	149.62 (8)	O12—Ag20—O30^{i}	102.6 (2)
S18—Ag8—O8	111.59 (15)	O12—Ag20—S4	123.9 (2)
S18—Ag8—O31	81.95 (15)	O30^{i}—Ag20—O7^{i}	83.0 (2)
O18—Ag9—O21	107.1 (3)	O30^{i}—Ag20—S4	124.34 (17)
O18—Ag9—S3	118.0 (2)	S4—Ag20—O7^{i}	134.42 (15)
O18—Ag9—S9	95.6 (2)	O1—Ag21—O12	100.8 (2)
O21—Ag9—S9	84.2 (2)	O1—Ag21—S1	115.19 (17)
S3—Ag9—O21	108.9 (2)	O12—Ag21—S1	128.97 (18)
S3—Ag9—S9	136.45 (9)	O13—Ag21—O1	99.4 (3)
S3—Ag10—O11	109.76 (15)	O13—Ag21—O12	87.2 (3)
S5—Ag10—O11	87.72 (15)	O13—Ag21—S1	118.9 (2)
Symmetry code: (i) −*x*+3/2, −*y*+3/2, *z*.			
**SD/Ag12**			
Ag1—Ag2	3.269 (2)	Ag17—S18	2.467 (3)
Ag1—Ag3	3.306 (2)	Ag18—Ag19	3.0057 (17)
Ag1—Ag4	3.3423 (17)	Ag18—O11	2.565 (11)
Ag1—O21	2.293 (18)	Ag18—S18	2.463 (4)
Ag1—S20	2.453 (4)	Ag18—S19	2.460 (4)
Ag1—S32	2.488 (4)	Ag19—Ag20	3.2169 (17)
Ag2—Ag8	3.3535 (14)	Ag19—O10	2.54 (2)
Ag2—Ag18	3.2298 (17)	Ag19—O33	2.538 (14)
Ag2—O19	2.411 (18)	Ag19—S7	2.494 (4)
Ag2—S19	2.437 (4)	Ag19—S19	2.536 (4)
Ag2—S32	2.483 (4)	Ag20—O32	2.488 (12)
Ag3—N1	2.364 (19)	Ag20—O45	2.475 (18)

Ag3—S19	2.511 (5)	Ag20—S7	2.511 (4)
Ag3—S20	2.479 (4)	Ag20—S14	2.544 (4)
Ag4—Ag5	3.2921 (15)	Ag21—Ag22	3.2297 (18)
Ag4—Ag34	3.0102 (16)	Ag21—Ag35	3.2144 (17)
Ag4—Ag35	2.9822 (16)	Ag21—S12	2.446 (3)
Ag4—O12	2.469 (18)	Ag21—S14	2.449 (3)
Ag4—S20	2.508 (3)	Ag22—Ag23	3.0336 (17)
Ag4—S21	2.472 (3)	Ag22—Ag24	2.9744 (15)
Ag5—Ag7	3.2876 (16)	Ag22—O43	2.527 (15)
Ag5—S21	2.500 (3)	Ag22—S4	2.495 (4)
Ag5—S30	2.534 (3)	Ag22—S12	2.509 (4)
Ag6—Ag29	3.2240 (18)	Ag23—Ag24	3.045 (2)
Ag6—Ag31	3.2117 (17)	Ag23—O16	2.513 (15)
Ag6—S23	2.437 (3)	Ag23—S4	2.460 (3)
Ag6—S30	2.445 (3)	Ag23—S27	2.483 (4)
Ag7—Ag8	2.9561 (18)	Ag24—O18	2.517 (12)
Ag7—Ag31	2.9906 (14)	Ag24—S12	2.460 (3)
Ag7—O12	2.465 (12)	Ag24—S27	2.476 (4)
Ag7—O36	2.568 (12)	Ag25—Ag26	3.2304 (17)
Ag7—S30	2.430 (3)	Ag25—O40	2.414 (14)
Ag7—S32	2.449 (3)	Ag25—S12	2.562 (4)
Ag8—Ag9	3.1902 (19)	Ag25—S15	2.516 (4)
Ag8—Ag31	2.9849 (13)	Ag26—Ag28	3.0776 (18)
Ag8—O11	2.430 (12)	Ag26—Ag29	2.9978 (18)
Ag8—O22	2.534 (16)	Ag26—O18	2.569 (13)
Ag8—S31	2.479 (3)	Ag26—S10	2.465 (4)
Ag8—S32	2.497 (4)	Ag26—S15	2.444 (4)
Ag9—O23	2.422 (16)	Ag27—Ag32	3.0441 (18)
Ag9—O25	2.469 (17)	Ag27—Ag33	3.260 (2)
Ag9—S18	2.558 (3)	Ag27—O51	2.528 (16)
Ag9—S31	2.541 (3)	Ag27—O52	2.41 (2)
Ag10—O27	2.496 (12)	Ag27—S10	2.478 (4)
Ag10—O59	2.451 (9)	Ag27—S27	2.489 (4)
Ag10—S24	2.573 (3)	Ag28—Ag29	3.0605 (17)
Ag10—S31	2.516 (3)	Ag28—O17	2.466 (13)
Ag11—Ag12	3.2499 (14)	Ag28—O57	2.559 (11)
Ag11—Ag17	3.2372 (15)	Ag28—S10	2.491 (4)
Ag11—S8	2.453 (3)	Ag28—S23	2.489 (3)
Ag11—S18	2.451 (3)	Ag29—S15	2.475 (4)
Ag12—Ag13	2.9590 (16)	Ag29—S23	2.481 (3)
Ag12—Ag14	3.0524 (15)	Ag30—O58	2.502 (10)
Ag12—S8	2.467 (3)	Ag30—S23	2.535 (4)

Ag12—S24	2.470 (3)	Ag30—S24	2.495 (4)
Ag13—Ag14	3.0331 (16)	Ag31—O60	2.561 (9)
Ag13—O48	2.535 (16)	Ag31—S30	2.519 (4)
Ag13—S3	2.455 (3)	Ag31—S31	2.481 (4)
Ag13—S8	2.451 (3)	Ag32—Ag33	3.0872 (18)
Ag14—Ag30	3.1399 (17)	Ag32—O49	2.423 (12)
Ag14—O17	2.548 (15)	Ag32—O55	2.411 (10)
Ag14—S3	2.491 (4)	Ag32—S3	2.471 (4)
Ag14—S24	2.479 (3)	Ag32—S10	2.505 (4)
Ag15—Ag23	3.1349 (18)	Ag33—O50	2.336 (18)
Ag15—O30	2.379 (17)	Ag33—S3	2.471 (4)
Ag15—O47	2.525 (15)	Ag33—S27	2.483 (4)
Ag15—S4	2.516 (4)	Ag34—Ag35	3.0552 (14)
Ag15—S8	2.592 (3)	Ag34—S14	2.449 (3)
Ag16—O29	2.477 (12)	Ag34—S20	2.470 (4)
Ag16—O44	2.519 (12)	Ag35—O41	2.510 (16)
Ag16—S4	2.548 (3)	Ag35—S14	2.495 (3)
Ag16—S7	2.530 (3)	Ag35—S21	2.487 (4)
Ag17—Ag18	3.0357 (16)	Ag36—O37	2.490 (13)
Ag17—Ag19	2.9526 (16)	Ag36—O42	2.393 (13)
Ag17—O28	2.46 (2)	Ag36—S15	2.637 (3)
Ag17—S7	2.449 (3)	Ag36—S21	2.593 (3)
O21—Ag1—S20	121.0 (5)	O21—Ag1—S32	94.1 (6)
S20—Ag1—S32	138.11 (14)	N1—Ag3—S19	113.2 (6)
N1—Ag3—S20	113.5 (6)	S20—Ag3—S19	132.84 (12)
O12—Ag4—S20	96.4 (4)	S21—Ag4—O12	104.2 (4)
S21—Ag4—S20	155.74 (13)	S21—Ag5—S30	155.33 (12)
S23—Ag6—S30	163.32 (12)	O12—Ag7—O36	87.1 (5)
S30—Ag7—O12	102.2 (4)	S30—Ag7—O36	107.2 (3)
S30—Ag7—S32	161.52 (13)	S32—Ag7—O12	91.0 (4)
S32—Ag7—O36	86.2 (3)	O11—Ag8—O22	102.6 (5)
O11—Ag8—S31	109.7 (3)	O11—Ag8—S32	95.7 (4)
S31—Ag8—O22	95.8 (7)	S31—Ag8—S32	151.52 (12)
S32—Ag8—O22	90.9 (7)	O23—Ag9—O25	110.1 (6)
O23—Ag9—S1	103.0 (5)	O23—Ag9—S31	93.1 (5)
O25—Ag9—S18	89.7 (3)	O25—Ag9—S31	103.5 (3)
S31—Ag9—S18	154.26 (12)	O27—Ag10—S24	92.8 (3)
O27—Ag10—S31	97.7 (3)	O59—Ag10—O27	102.7 (4)
O59—Ag10—S24	92.9 (2)	O59—Ag10—S31	103.1 (2)
S31—Ag10—S24	158.33 (10)	S18—Ag11—S8	155.08 (13)
S8—Ag12—S24	162.46 (12)	S3—Ag13—O48	95.3 (4)
S8—Ag13—O48	94.8 (5)	S8—Ag13—S3	165.90 (12)

S3—Ag14—O17	86.6 (4)	S24—Ag14—O17	106.2 (4)
S24—Ag14—S3	157.66 (14)	O30—Ag15—O47	90.7 (9)
O30—Ag15—S4	108.0 (4)	O30—Ag15—S8	87.3 (4)
O47—Ag15—S8	89.2 (4)	S4—Ag15—O47	111.2 (4)
S4—Ag15—S8	153.71 (13)	O29—Ag16—O44	104.9 (4)
O29—Ag16—S4	97.6 (3)	O29—Ag16—S7	98.5 (3)
O44—Ag16—S4	90.9 (3)	O44—Ag16—S7	99.3 (3)
S7—Ag16—S4	157.98 (11)	O28—Ag17—S18	96.9 (5)
S7—Ag17—O28	96.2 (6)	S7—Ag17—S18	165.35 (13)
S18—Ag18—O11	91.8 (3)	S19—Ag18—O11	95.0 (3)
S19—Ag18—S18	161.72 (13)	O33—Ag19—O10	101.0 (6)
S7—Ag19—O10	101.1 (5)	S7—Ag19—O33	105.2 (4)
S7—Ag19—S19	155.79 (14)	S19—Ag19—O10	98.2 (5)
S19—Ag19—O33	85.2 (4)	O32—Ag20—S7	98.4 (4)
O32—Ag20—S14	99.0 (4)	O45—Ag20—O32	102.0 (7)
O45—Ag20—S7	104.2 (5)	O45—Ag20—S14	92.2 (6)
S7—Ag20—S14	153.00 (11)	S12—Ag21—S14	157.46 (12)
S4—Ag22—O43	95.2 (5)	S4—Ag22—S12	162.34 (11)
S12—Ag22—O43	102.2 (5)	S4—Ag23—O16	106.2 (4)
S4—Ag23—S27	153.82 (14)	S27—Ag23—O16	93.5 (4)
S12—Ag24—O18	97.1 (4)	S12—Ag24—S27	162.62 (14)
S27—Ag24—O18	91.9 (4)	O40—Ag25—S12	97.5 (4)
O40—Ag25—S15	106.6 (4)	S15—Ag25—S12	152.24 (13)
S10—Ag26—O18	87.2 (4)	S15—Ag26—O18	107.9 (4)
S15—Ag26—S10	158.38 (13)	O52—Ag27—O51	85.9 (7)
O52—Ag27—S10	105.3 (5)	O52—Ag27—S27	104.3 (6)
S10—Ag27—O51	115.9 (4)	S10—Ag27—S27	135.72 (12)
S27—Ag27—O51	98.4 (4)	O17—Ag28—O57	100.5 (5)
O17—Ag28—S10	90.9 (4)	O17—Ag28—S23	97.0 (4)
S10—Ag28—O57	112.8 (3)	S23—Ag28—O57	84.7 (3)
S23—Ag28—S10	159.25 (13)	S15—Ag29—S23	162.65 (13)
O58—Ag30—S23	92.8 (3)	S24—Ag30—O58	103.7 (3)
S24—Ag30—S23	158.67 (10)	S30—Ag31—O60	89.5 (3)
S31—Ag31—O60	106.3 (3)	S31—Ag31—S30	163.41 (10)
O49—Ag32—S3	108.4 (5)	O49—Ag32—S10	98.5 (5)
O55—Ag32—O49	91.3 (4)	O55—Ag32—S3	106.4 (3)
O55—Ag32—S10	95.8 (3)	S3—Ag32—S10	144.32 (11)
O50—Ag33—S3	105.9 (5)	O50—Ag33—S27	100.0 (6)
S3—Ag33—S27	139.68 (14)	S14—Ag34—S20	163.11 (13)
S14—Ag35—O41	92.6 (4)	S21—Ag35—O41	103.7 (4)
S21—Ag35—S14	162.73 (12)	O37—Ag36—S15	91.8 (3)
O37—Ag36—S21	102.2 (3)	O42—Ag36—O37	102.7 (4)

Supplementary Methods.

All chemicals and solvents used in the syntheses were of analytical grade and used without further purification. ^{i}PrSH (Adamas-beta®) was purchased from Shanghai Titan Scientific Co., Ltd. IR spectra were recorded on a PerkinElmer Spectrum Two in the frequency range of 4000-450 cm^{-1}. The elemental analyses (C, H, N contents) were determined on a Vario EL III analyzer. The diffuse-reflectance spectra were performed on UV−Vis spectrophotometer (Evolution 220, ISA-220 accessory, Thermo Scientific) using a built-in 10 mm silicon photodiode with a 60 mm Spectralon sphere. The emission spectra were investigated on a Lumina fluorescence spectrometer (Thermo Scientific). ^{13}C NMR spectrum was recorded in a J. Young NMR tube on Bruker Avance 500 spectrometers. The chemical shifts are reported in parts per million δ (ppm) referenced to the residual proton signal of the deuterated methanol. Powder X-ray diffraction (PXRD) data were collected on a Philips X'Pert Pro MPD X-ray diffractometer with CuKα radiation equipped with an X'Celerator detector. Energy-dispersive X-ray spectrum was measured using an SU-8010 field emission scanning electron microscope (FESEM; Hitachi Ltd., Tokyo, Japan) equipped with an Oxford-Horiba Inca XMax50 energy-dispersive X-ray (EDX; Oxford Instruments Analytical, High Wycombe, England). Mass spectra were recorded on an Agilent 6224 (Agilent Technologies, USA) ESI-TOF-MS spectrometer. Sample solutions are infused by a syringe pump at 4 μL/min. Data were acquired using the following settings: ESI capillary voltage was set at 4000 V (+) ion mode and 3500 V (−) ion mode and fragmentor at 200 V. The liquid nebulizer was set to 15 psig and the nitrogen drying gas was set to a flow rate of 4 L/min. Drying gas temperature was maintained at 150 °C. The data analyses of mass spectra were performed based on the isotope distribution patterns using Agilent MassHunter Workstation Data acquisition software (Version B.05.00). The reported *m/z* values represent monoisotopic mass of the most abundant peak (100%) within the isotope pattern.

Supplementary Note 1.

Single crystals of **SD/Ag7-SD/Ag12** with appropriate dimensions were chosen under an optical microscope and quickly coated with high vacuum grease (Dow Corning Corporation) to prevent decomposition. Intensity data and cell parameters were recorded at 123K for **SD/Ag7** and 100K for **SD/Ag8** and **SD/Ag12** on a Bruker Apex II single crystal diffractometer, employing Mo K_{α} radiation (λ = 0.71073 Å) and a CCD area detector. The raw frame data were processed using SAINT and SADABS to yield the reflection data file.[1] Single-crystal X-ray diffraction data of **SD/Ag9-SD/Ag11** were collected on a Rigaku Oxford Diffraction XtaLAB Synergy diffractometer equipped with a HyPix-6000HE area detector at 100 K using Mo Kα (λ = 0.71073 Å) from PhotonJet micro-focus X-ray Source. The structure was solved using the charge-flipping algorithm, as implemented in the program *SUPERFLIP*[2] and refined by full-matrix least-squares techniques against F_o^2 using the SHELXL program[3] through the OLEX2 interface.[4] Hydrogen atoms at carbon were placed in calculated positions and refined isotropically by using a riding model. Appropriate restraints or constraints were applied to the geometry and the atomic displacement parameters of the atoms in the cluster. All structures were examined using the Addsym subroutine of PLATON[5] to ensure that no additional symmetry could be applied to the models. Pertinent crystallographic data collection and refinement parameters are collated in Supplementary Table 1. Selected bond lengths and angles are collated in Supplementary Table 6.

Supplementary References:

1 *Bruker AXS, APEX2, V2008.6; SADABS V2008/1; SAINT V7.60A; SHELXTL V6.14*; Bruker AXS Inc.: Madison, Wisconsin, USA, **2008**

2 Palatinus, L. & Chapuis, G. Superflip- a computer program for the solution of crystal structures by charge flipping in arbitrary dimensions. *J Appl Crystallogr* **40**, 786-790, (2007).

3 Sheldrick, G. M. Crystal structure refinement with Shelxl. *Acta Crystallogr C* **71**, 3-8, (2015).

4 Dolomanov, O. V., Bourhis, L. J., Gildea, R. J., Howard, J. A. K. & Puschmann, H. Olex2: a complete structure solution, refinement and analysis program. *J Appl Crystallogr* **42**, 339-341, (2009).

5 Spek, A. L. Structure validation in chemical crystallography. *Acta Crystallogr D* **65**, 148-155, (2009).
